# Targeting photodynamic and photothermal therapy to the endoplasmic reticulum enhances immunogenic cancer cell death

**DOI:** 10.1038/s41467-019-11269-8

**Published:** 2019-07-26

**Authors:** Wei Li, Jie Yang, Lihua Luo, Mengshi Jiang, Bing Qin, Hang Yin, Chunqi Zhu, Xiaoling Yuan, Junlei Zhang, Zhenyu Luo, Yongzhong Du, Qingpo Li, Yan Lou, Yunqing Qiu, Jian You

**Affiliations:** 1 College of Pharmaceutical Sciences, Zhejiang University, 866 Yuhangtang Road, 310058 Hangzhou, Zhejiang P. R. China; 2 The First Affiliated Hospital of Medical School of Zhejiang University, 79 Qingchun Road, 310058 Hangzhou, Zhejiang P. R. China

**Keywords:** Cancer microenvironment, Cancer immunotherapy

## Abstract

Immunogenic cell death (ICD)-associated immunogenicity can be evoked through reactive oxygen species (ROS) produced via endoplasmic reticulum (ER) stress. In this study, we generate a double ER-targeting strategy to realize photodynamic therapy (PDT) photothermal therapy (PTT) immunotherapy. This nanosystem consists of ER-targeting pardaxin (FAL) peptides modified-, indocyanine green (ICG) conjugated- hollow gold nanospheres (FAL-ICG-HAuNS), together with an oxygen-delivering hemoglobin (Hb) liposome (FAL-Hb lipo), designed to reverse hypoxia. Compared with non-targeting nanosystems, the ER-targeting naosystem induces robust ER stress and calreticulin (CRT) exposure on the cell surface under near-infrared (NIR) light irradiation. CRT, a marker for ICD, acts as an ‘eat me’ signal to stimulate the antigen presenting function of dendritic cells. As a result, a series of immunological responses are activated, including CD8^+^ T cell proliferation and cytotoxic cytokine secretion. In conclusion, ER-targeting PDT-PTT promoted ICD-associated immunotherapy through direct ROS-based ER stress and exhibited enhanced anti-tumour efficacy.

## Abstract (web summary)

Reactive oxygen species induced by endoplasmic reitculum stress can be exploited for cancer therapy. Here, nanoparticles are targetted to the endoplasmic reticulum and, when accompanied by PDT, produce stress resulting in calreticulin exposure on the cell surface, which activates dendritic cells.

## Introduction

Solid tumors maintain an immune-dysfunctional microenvironment by displaying low immunogenicity, secreting immunosuppressive cytokines, and dying through tolerogenic pathways^1,2^. Various therapy modalities such as chemotherapy^3^, radiotherapy^4^, and (PDT)^5^ have been applied to induce immunogenic cell death (ICD). Damage-associated molecular patterns (DAMPs) such as calreticulin (CRT), heat shock proteins (HSP70 and HSP90), and high mobility group box 1 (HMGB1) could be induced after ICD^6–8^. These danger signals activate the host immune system against cancer by stimulating antigen presentation of dendritic cells (DCs) and proliferation of cytotoxic T lymphocyte (CD8^+^ T cells)^6–8^.

Endoplasmic reticulum (ER), where CRT locates, plays a crucial role in the maintenance of intracellular signal transduction, calcium homeostasis, protein synthesis, and processing^9^. ER stress and reactive oxygen species (ROS) production are essential for activating intracellular signaling pathways that govern ICD^10,11^. Although certain chemotherapeutic agents (e.g., doxorubicin and mitoxantrone) and radiotherapy can induce secondary or collateral ER stress effects^7,10,12,13^, it is more effective to promote ICD-associated immunogenicity through ER-directed and ROS-based ER stress^7,10,12,13^. It has been reported that hypericin-induced PDT can produce high ROS level in ER by ER-targeted accumulation of the photosensitizer^13,14^, thus causing strong ICD. However, most photosensitizers do not have the ability to accumulate into ER (e.g., indocyanine green (ICG), mainly distributing in the cytoplasm after cellular internalization^15^), which limits the occurrence of strong ICD under illumination. Thus ER-localized PDT might fit the criterion of primary ER-directed ROS production, presenting a therapeutic modality for ICD-associated immunotherapy.

One drawback with PDT is its oxygen-consuming process^16^. Low oxygen levels, in turn, severely limit production of ROS in PDT, thus weakening ROS-based ER stress and ICD effects^17^. Here we constructed a combined PTT/PDT nanosystem by conjugating ICG on hollow gold nanospheres (HAuNS), which was further modified by FAL peptides to endow it the ER-targeting ability. Hemoglobin (Hb), an efficient and safe oxygen carrier that releases oxygen under hypoxia^18,19^, was encapsulated in FAL-modified liposomes to act as an assist of PDT. The obtained FAL-ICG-HAuNS and FAL-Hb-lipo both displayed ER-specific accumulation after cell internalization and increased intracellular stability by avoiding the enzymes in lysosomes. NIR light (808 nm) mediated PTT/PDT in ER under sufficient oxygen supply induced robust ROS-based ER stress, characterized by upregulation of CHOP (C/EBP-homologous protein-10; an ER apoptotic protein). Subsequently, the specific ER stress led to effective ER-to-cell surface translocation of CRT, which was indicative of ICD^20^. CRT exposure acted as an “eat me” signal, stimulating naive DCs to mature and inducing enhanced immune responses. Prominent inhibition of tumor growth and prolonged animal survival were achieved as a result of synchronous ER-targeting PTT/PDT/immunotherapy. Thus we have built double “ER missiles”, which could be used not only for PTT/PDT but also for ICD-associated cancer immunotherapy through induction of specific ER stress under the control of near infrared (NIR) light.

## Results

### Characterization of four types of nanospheres

**Fig. 1:**
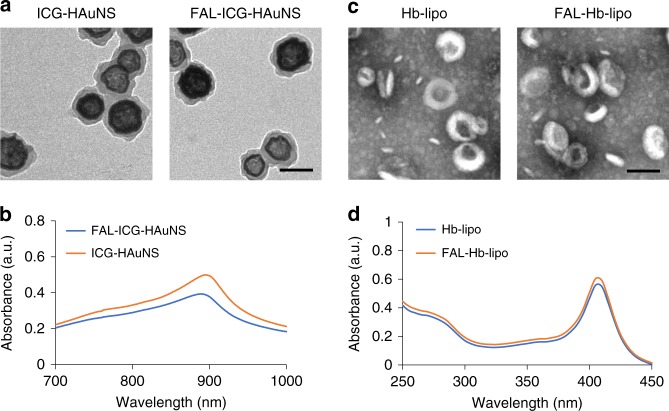
Characterization. **a** Representative transmission electron microscopic (TEM) images of ICG-HAuNS and FAL-ICG-HAuNS. Scale bars, 100 nm. **b** Ultraviolet–visible (UV-vis) absorption spectra of ICG-HAuNS and FAL-ICG-HAuNS (indocyanine green (ICG): 10 μg/mL; hollow gold nanospheres (HAuNS): 20 μg/mL). **c** Representative TEM images of Hb-lipo and FAL-Hb-lipo. Scale bars, 100 nm. **d** UV-vis absorption spectra of Hb-lipo and FAL-Hb-lipo (hemoglobin (Hb): 80 μg/mL)

As an excellent photothermal reagent in the NIR region, HAuNS displayed a maximum absorption peak around 780–800 nm (Supplementary Fig. 1). To enable ER targeting of ICG-HAuNS, a peptide named FAL was conjugated to the particle using NH_2_-PEG-TA as a linker. ^1^H nuclear magnetic resonance was performed to verify the chemical structures of different compounds. The characteristic peak of -COOH on thioctic acid (TA) at about 12.0 ppm disappeared, while the peaks of PEG and FAL appeared in the spectrum of FAL-PEG-HAuNS (Supplementary Fig. 2). The results indicated successful conjugation of FAL-PEG-TA onto HAuNS. Both ICG-HAuNS and FAL-ICG-HAuNS presented with a membrane structure outside the hollow gold shell as observed by transmission electron microscopy (TEM; Fig. 1a). The hydrated particle sizes of ICG-HAuNS and FAL-ICG-HAuNS were 122 ± 6.5 and 151 ± 4.6 nm, respectively, as measured by dynamic light scattering. The absorption peaks of ICG had a red shift to around 890 nm after the chemical conjugation with HAuNS (Fig. 1b). ICG contents in ICG-HAuNS and FAL-ICG-HAuNS were 50.5 ± 3.7% and 49.3 ± 6.1%, respectively.

As presented in the TEM images, both Hb-lipo and FAL-Hb-lipo displayed a spherical structure with homogeneous lipid bilayer (Fig. 1c), and the particle sizes were 153.2 ± 7.8 and 160.5 ± 6.6 nm, respectively. In addition, Hb had an absorption peak at about 400 nm as scanned by an ultraviolet–visible (UV-vis) spectrophotometer (Fig. 1d). The Hb contents of Hb-lipo and FAL-Hb-lipo were determined to be 64.6 ± 5% and 60.8 ± 7.8%, respectively.

### Subcellular localization and cell internalization

**Fig. 2:**
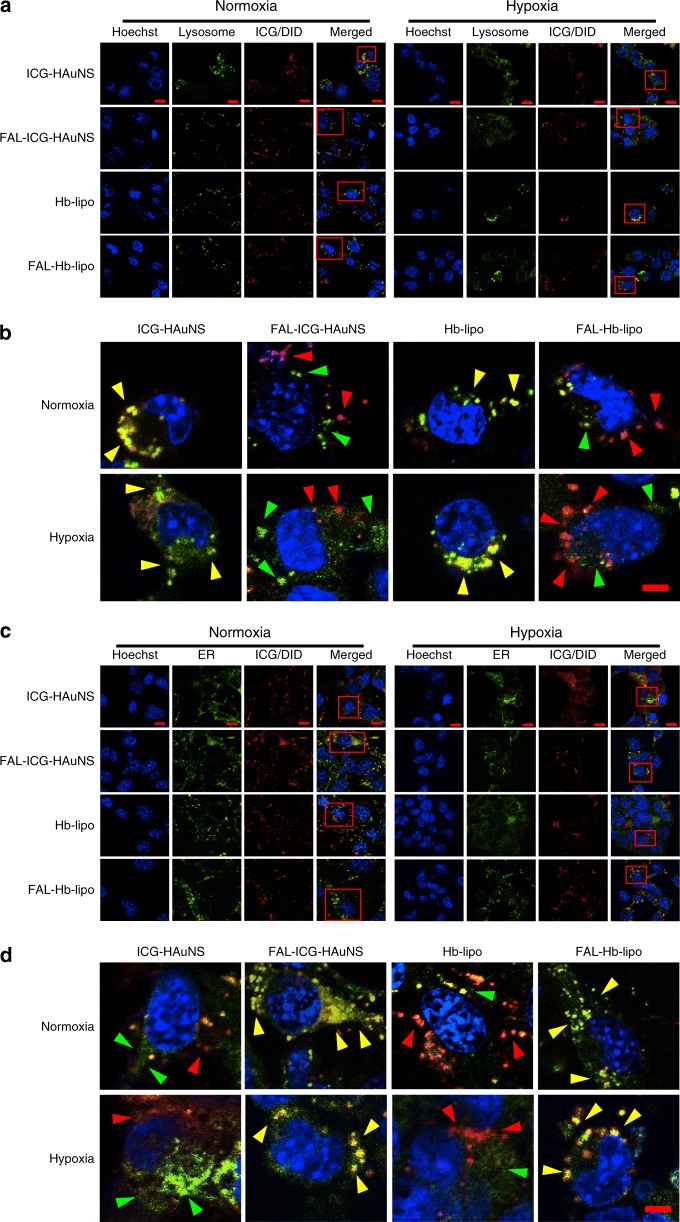
Lysosome and endoplasmic reticulum (ER) co-localization. **a** Representative fluorescent images of lysosomal co-localization of ICG-HAuNS, FAL-ICG-HAuNS, Hb-lipo, or FAL-Hb-lipo under normoxia and hypoxia, respectively. Scale bars, 10 μm, *n* = 3. **b** Enlarged images of **a** (red boxes). Yellow arrows indicate “merge”; green arrows indicate “lysosome”; red arrows indicate “ICG/DID”. Scale bars, 5 μm, *n* = 3. **c** Representative fluorescent images of ER co-localization of ICG-HAuNS, FAL-ICG-HAuNS, Hb-lipo, or FAL-Hb-lipo under normoxia and hypoxia, respectively. Scale bars, 10 μm, *n* = 3. **d** Enlarged images of **c** (red boxes). Yellow arrows indicate “merge”; green arrows indicate “ER”; red arrows indicate “ICG/DID”. Scale bars, 5 μm, *n* = 3

To evaluate ER-targeting ability mediated by FAL peptides, lysosomes and ER were stained, respectively, and subcellular localization of ICG-HAuNS, FAL-ICG-HAuNS, Hb-lipo, or FAL-Hb-lipo was examined under normoxia and hypoxia conditions. As shown in Fig. 2a–d, ICG-HAuNS and Hb-lipo were mostly co-localized with lysosomes regardless of the culture condition. However, the fluorescent FAL-ICG-HAuNS and FAL-Hb-lipo particles aligned with ER instead of lysosomes, which should be attributed to the special affinity between FAL peptides and ER.

Cell internalization of particles was studied by detecting ICG or DiD fluorescence inside the cancer cells. Uptake rate of ICG-HAuNS was slightly higher than that of Hb-lipo, especially at 24 h (Supplementary Fig. 3). The intracellular fluorescence of FAL-ICG-HAuNS and FAL-Hb-lipo was stronger than that of ICG-HAuNS and Hb-lipo, respectively. A possible explanation was that the ER-targeting nanospheres, which could escape from the influence of various hydrolytic enzymes in the lysosomes, had a better intracellular stability. The amount of ICG-HAuNS in CT-26 cells decreased about 60% at 24 h, while 62.6% FAL-ICG-HAuNS could still be detected under the same circumstance (Supplementary Fig. 4). The high stability of FAL-modified nanospheres was a prerequisite for subsequent antitumor effect.

### ROS generation and antitumor effect in vitro

**Fig. 3:**
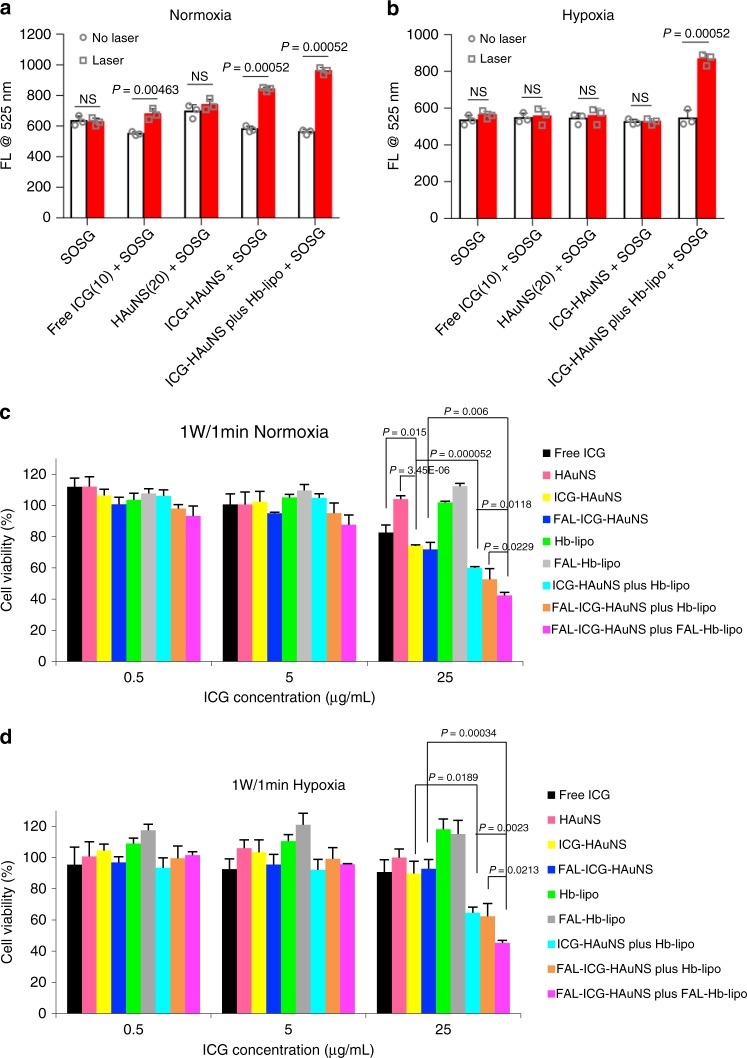
Reactive oxygen species (ROS) generation and antitumor effect in vitro. **a** ROS generation in free indocyanine green (ICG; 10 μg/mL), hollow gold nanospheres (HAuNS; 20 μg/mL), ICG-HAuNS (ICG: 10 μg/mL; HAuNS: 20 μg/mL), or ICG-HAuNS plus Hb-lipo (ICG: 10 μg/mL; HAuNS: 20 μg/mL; Hb: 20 μg/mL) treatment groups under normoxia detected with Singlet Oxygen Sensor Green (SOSG). Laser power: 1 W/cm^2^, 10 min, *n* = 3. **b** ROS generation in free ICG (10 μg/mL), HAuNS (20 μg/mL), ICG-HAuNS (ICG: 10 μg/mL; HAuNS: 20 μg/mL), or ICG-HAuNS plus Hb-lipo (ICG: 10 μg/mL; HAuNS: 20 μg/mL; Hb: 20 μg/mL) treatment groups under hypoxia detected with SOSG. Laser power: 1 W/cm^2^, 10 min, *n* = 5. **c**, **d** CT-26 cell viability after incubation with free ICG, HAuNS, ICG-HAuNS, FAL-ICG-HAuNS, Hb-lipo, FAL-Hb-lipo, ICG-HAuNS plus Hb-lipo, FAL-ICG-HAuNS plus Hb-lipo, or FAL-ICG-HAuNS plus FAL-Hb-lipo (equivalent ICG: 0.5, 5, 25 μg/mL; equivalent HAuNS: 1, 10, 50 μg/mL; equivalent Hb: 1, 10, 50 μg/mL) for 48 h under normoxia (**c**) or hypoxia (**d**), *n* = 5. All data were analyzed with one-way analysis of variance test. All error bars are expressed as ±SD. “NS” indicates “not significant”

To determine whether Hb-lipo could be used as a PDT adjuvant, single oxygen species (^1^O_2_)-producing capability under normal and hypoxia conditions was first evaluated by detecting changes in fluorescence from Singlet Oxygen Sensor Green (SOSG). When exposed to the same laser irradiation, ICG-HAuNS induced a significant SOSG fluorescence enhancement. With the help of oxygen-delivering Hb-lipo, the amount of ^1^O_2_ could be further elevated (Fig. 3a). In addition, ^1^O_2_ generation was completely inhibited under hypoxia except in the ICG-HAuNS plus Hb-lipo group (Fig. 3b). The oxygen-carrying Hb-lipo was able to promote ^1^O_2_ production even in the hypoxia environment. The results revealed that PDT was an oxygen-consuming procedure, and it could be maintained at a high efficiency only if there was sufficient oxygen supply.

Intracellular ROS generation was further evaluated in CT-26 cells using the 2′,7′-dichlorodihydrofluorescein diacetate (DCFH-DA) probe. Owing to the excellent intracellular stability (Supplementary Fig. 4), FAL-ICG-HAuNS induced higher ROS level than free ICG or ICG-HAuNS. Adding oxygen-delivering Hb-lipo enabled ICG-HAuNS to produce an enhanced level of ROS. Moreover, ROS production further increased when ICG-HAuNS and Hb-lipo were simultaneously imported into ER by FAL peptide (Supplementary Fig. 5a, b).

To investigate antitumor effect from the combined ER-targeting and oxygen-delivering nanosystem, phototoxicity of different drugs was evaluated with the 3-(4,5-dimethy lthiazol-2-yl)-2,5-diphenyltetra-zolium bromide (MTT) assay under normoxia or hypoxia. ICG-HAuNS induced more cell death than free ICG or HAuNS owing to the synchronous photothermal and photodynamic effect triggered by NIR light with the ICG concentration of 25 μg/mL under normoxia (Fig. 3c, d). Cytotoxicity from free ICG, HAuNS, and ICG-HAuNS decreased by about 20% under hypoxia. However, the ICG-HAuNS plus Hb-lipo group (Fig. 3c, d) still caused about 46% cell death with the ICG concentration of 25 μg/mL under hypoxia. The results indicated that the oxygen supplied by Hb played an important role in antitumor effect. Furthermore, FAL-ICG-HAuNS plus FAL-Hb-lipo showed more efficient cell killing than FAL-ICG-HAuNS plus Hb-lipo with the ICG concentration of 25 μg/mL (Fig. 3c, d), possibly due to the severe PTT/PDT effect from sufficient oxygen supply in ER. Surprisingly, when ICG concentration was at 25 μg/mL, cell survival in the FAL-ICG-HAuNS treatment group increased to 92.7% under hypoxia comparing to 71.8% under normoxia, while there was no change in cytotoxicity between hypoxia and normoxia conditions from the FAL-ICG-HAuNS plus Hb-lipo and FAL-ICG-HAuNS plus FAL-Hb-lipo treatment groups (Fig. 3c, d). A plausible explanation was that ER-targeting FAL-ICG-HAuNS induced intense ER stress, which could easily cause cell apoptosis with abundant oxygen supply. In addition, as a drug-delivering system with great biocompatibility, Hb-lipo and FAL-Hb-lipo showed little cytotoxicity.

### Specific ER stress and CRT exposure

ICD is a cell death mechanism characterized by upregulation of various DAMPs. Several studies have demonstrated a close relationship between ROS, ER stress, and ICD^10,21,22^. ICD-associated immunogenicity induced by direct ROS-based ER stress is more effective than secondary or collateral ER stress effects.

**Fig. 4:**
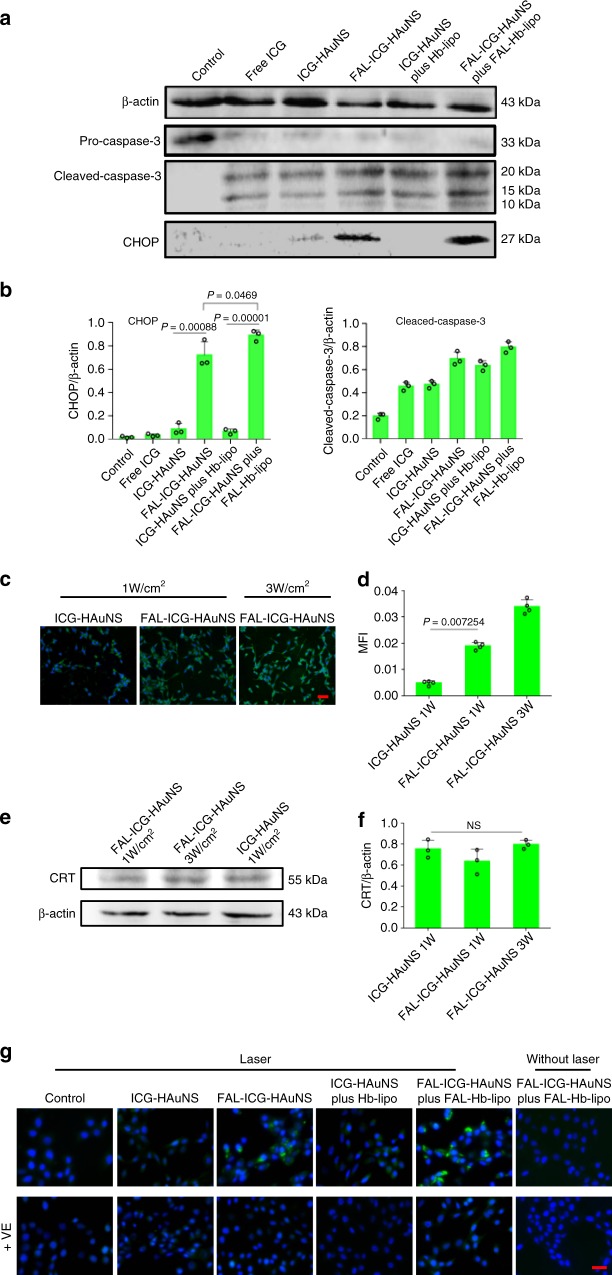
Specific endoplasmic reticulum stress and calreticulin (CRT) exposure. **a** Western blot analysis of caspase-3 and CHOP (C/EBP-homologous protein-10) proteins expressed in CT-26 cells after treatment with free indocyanine green (ICG; 20 μg/mL), ICG-HAuNS (ICG: 20 μg/mL; hollow gold nanospheres (HAuNS): 40 μg/mL), FAL-ICG-HAuNS (ICG: 20 μg/mL; HAuNS: 40 μg/mL), ICG-HAuNS (ICG: 20 μg/mL; HAuNS: 40 μg/mL) plus Hb-lipo (40 μg/mL), or FAL-ICG-HAuNS (ICG: 20 μg/mL; HAuNS: 40 μg/mL) plus FAL-Hb-lipo (40 μg/mL) for 12 h. Laser power: 2 W/cm^2^, 2 min, *n* = 3. **b** Quantification of cleaved caspase-3 and CHOP proteins normalized by β-actin, respectively. **c**–**f** CRT exposure (**c**, **d**) and total amount of CRT (**e**, **f**) after treatment with ICG-HAuNS or FAL-ICG-HAuNS followed by laser irradiation (1 W/cm^2^ or 3 W/cm^2^, 2 min), *n* = 3. Ex: 488 nm. Scale bars, 50 μm. **g** Representative fluorescent imaging on CRT exposure with (1 W/cm^2^, 2 min) or without laser irradiation. Tocopherol (Vitamin E) was employed as a reactive oxygen species scavenger. Ex: 488 nm. Scale bars, 20 μm, *n* = 3. All data were analyzed with one-way analysis of variance test. All error bars are expressed as ±SD. “NS” indicates “not significant”

To further investigate specific ER stress induced by the FAL-ICG-HAuNS plus FAL-Hb-lipo, we applied western blot to examine cell apoptosis mechanism. CHOP is a pro-apoptotic protein overexpressed after ER stress^23,24^. Treatment of cells with free ICG, ICG-HAuNS, or ICG-HAuNS plus Hb-lipo followed by laser exposure had little effect CHOP activation (Fig. 4a, b). In contrast, FAL-ICG-HAuNS and FAL-ICG-HAuNS plus FAL-Hb-lipo caused a remarkable upregulation of CHOP proteins under laser irradiation, indicating that ER-targeting PDT could cause a more severe ER stress and ER-related cell apoptosis than non-targeting PDT. Caspase-3, a mitochondria-apoptotic protein, was activated in all the treatment groups, and FAL-ICG-HAuNS plus FAL-Hb-lipo induced the highest level of cleaved-caspase 3 (Fig. 4a, b). The results demonstrate that there was a crosstalk between ER stress and mitochondria and ER stress was capable of inducing mitochondria-related cell death.

Translocation of CRT from ER to cell surface is an indicator of ER stress response and a signal for ICD. We measured the amount of membrane-associated CRT and total amount of CRT in cells. CRT exposure on membrane was closely related to the intracellular localization of particles and the laser power (Fig. 4c, d). ER-targeting FAL-ICG-HAuNS induced a significant CRT exposure (ecto-CRT) compared with non-targeting ICG-HAuNS under the laser density of 1 W/cm^2^. In comparison, total amount of CRT protein remained constant regardless of laser treatment (Fig. 4e, f). The results demonstrated that the ER-targeting PDT could effectively promote CRT translocation.

To further study the role of ROS in ER stress induced by FAL-ICG-HAuNS, we employed Vitamin E (VE) as an ROS scavenger^25^. Similar to oxaliplatin treatment, FAL-ICG-HAuNS and FAL-ICG-HAuNS plus FAL-Hb-lipo treatment groups after laser irradiation showed a high level of CRT signal, while the other groups exhibited low CRT exposure (Fig. 4g, Supplementary Fig. 6). A plausible explanation was that ER-targeting ROS production under light treatment led to an increased level of ER stress response. As a result, the ER-located CRT trafficked to the cell surface. As a control, FAL-ICG-HAuNS plus FAL-Hb-lipo without laser treatment did not show obvious surface CRT level, indicating that ER stress could only be activated by NIR light-mediated PDT. CRT level on cell surface decreased after the ROS scavenger VE was added, indicating that ROS was the key for induction of ER stress.

### Biodistribution and antitumor effect in CT-26 tumor model

**Fig. 5:**
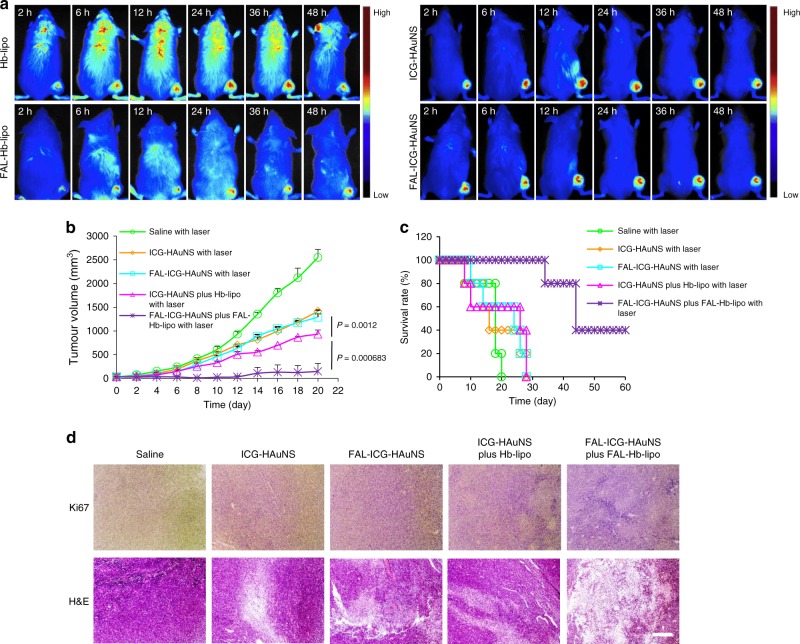
Biodistribution and antitumor effect in CT-26 tumor model. **a** Real-time in vivo fluorescent imaging in mice after they were intravenously injected with ICG-HAuNS (indocyanine green (ICG): 4 mg/kg; hollow gold nanospheres (HAuNS): 8 mg/kg), FAL-ICG-HAuNS (ICG: 4 mg/kg; HAuNS: 8 mg/kg), DiD@Hb-lipo (Hb: 20 mg/kg), or DiD@FAL-Hb-lipo (Hb: 20 mg/kg). Ex: 649 nm for DiD; 735 nm for ICG. **b** CT-26 tumor growth curves after treatment with saline, ICG-HAuNS (ICG: 0.5 mg/kg and HAuNS: 1 mg/kg per injection), FAL-ICG-HAuNS (ICG: 0.5 mg/kg and HAuNS: 1 mg/kg per injection), ICG-HAuNS (ICG: 0.5 mg/kg and HAuNS: 1 mg/kg per injection) plus Hb-lipo group (20 mg/kg per injection), or FAL-ICG-HAuNS (ICG: 0.5 mg/kg and HAuNS: 1 mg/kg per injection) plus FAL-Hb-lipo (20 mg/kg per injection). Laser power: 1 W/cm^2^, 2 min, *n* = 6. **c** Kaplan–Meier plots on animal survival, *n* = 6. **d** Representative Ki-67 and hematoxylin and eosin staining results in each group. Scale bars, 300 μm. All data were analyzed with one-way analysis of variance test. All error bars are expressed as ±SD

While both Hb-lipo and FAL-Hb-lipo could accumulate in tumors, ICG-HAuNS and FAL-ICG-HAuNS showed a higher tumor-targeting efficiency, demonstrating a clear ICG signal at 2 h postinjection (Fig. 5a). The biodistribution of the four types of particles was observed at 48 h. Owing to the rapid cell internalization and good stability, ICG-HAuNS and FAL-ICG-HAuNS exhibited a prominent tumor retention effect. It was also found that large amounts of Hb-lipo and FAL-Hb-lipo accumulated in the liver and spleen due to the recognition of reticuloendothelial system (Supplementary Fig. 7).

Tumor growth inhibition effect was also investigated. As displayed in Fig. 5b, CT-26 tumors grew rapidly in the saline group, in which the tumor size reached about 1000 mm^3^ after 12 days. ICG-HAuNS and FAL-ICG-HAuNS treatments had limited antitumor effect. Although NIR light-triggered PTT/PDT was able to inhibit tumor growth to a certain extent, tumors in these two groups still grew to about 1300 mm^3^ on day 20, which probably resulted from oxygen depletion after repeated PDT. Under the assist of Hb-lipo, ICG-HAuNS showed a slower tumor growth tendency thanks to the additionally provided oxygen. Moreover, the ER-targeting FAL-ICG-HAuNS plus FAL-Hb-lipo treatment exhibited a significantly enhanced antitumor effect under laser activation. Tumors in this group did not show significant growth in the first 12 days, which could be attributed to the intense ER stress via the ER-targeted PTT/PDT under sufficient oxygen supply. As a result, treatment with FAL-ICG-HAuNS plus FAL-Hb-lipo extended life span of the mice, and 40% mice lived through 60 days. In contrast, all the mice in the other four groups died within 30 days (Fig. 5c). Hematoxylin and eosin (H&E) and Ki-67 staining revealed severe damage to tumor tissue and low proliferation rate of tumor cell growth in the FAL-ICG-HAuNS plus FAL-Hb-lipo group (Fig. 5d). In addition, body weight of mice experienced a fluctuation except the FAL-ICG-HAuNS plus FAL-Hb-lipo group (Supplementary Fig. 8a). No obvious tissue damage was found in the H&E-stained pictures of the major organs (Supplementary Fig. 8b), demonstrating excellent biocompatibility of the combined therapy system.

### ICD induced by specific ER stress in CT-26 tumor model

**Fig. 6:**
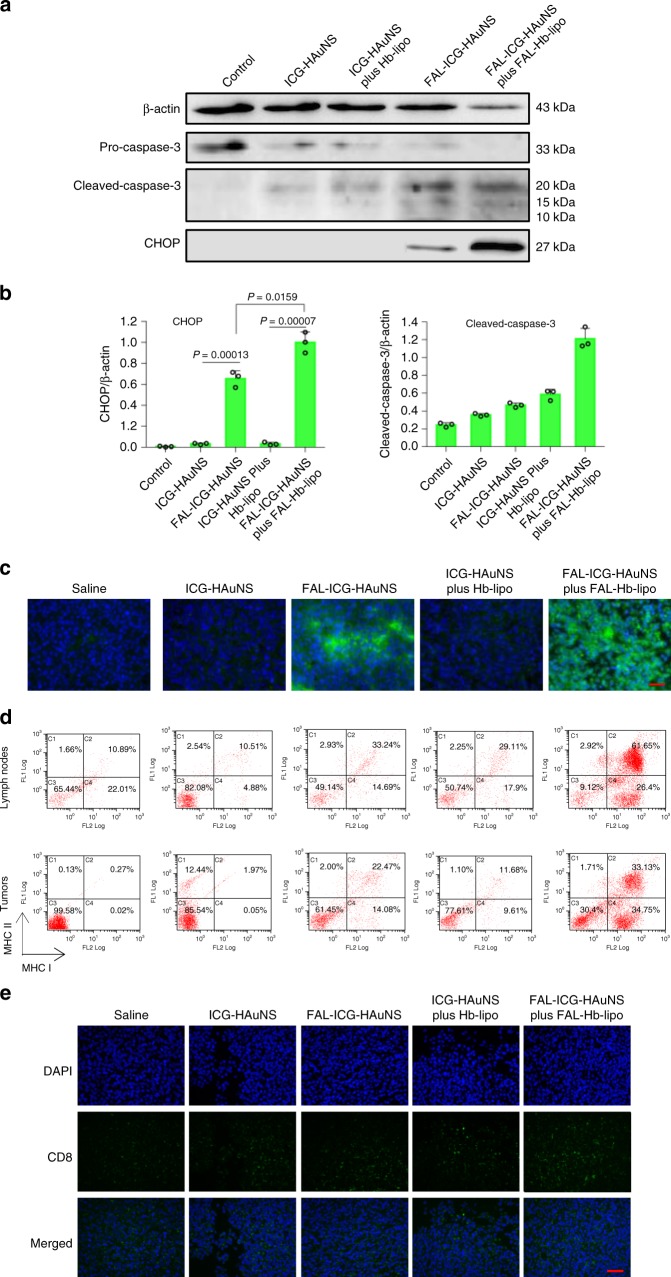
Immunogenic cell death induced by specific endoplasmic reticulum stress in CT-26 tumor model. Laser power: 1 W/cm^2^, 2 min. **a** Western blot analysis of caspase-3 and CHOP (C/EBP-homologous protein-10) proteins expressed in CT-26 tumors after drug administration and laser irradiation, *n* = 3. **b** Quantification of cleaved caspase-3 and CHOP proteins normalized by β-actin, respectively. **c** Representative fluorescent images of calreticulin expression in CT-26 tumor slices. Scale bars, 50 μm, *n* = 3. **d** Representative images of dendritic cell maturation in tumors and lymph nodes analyzed with flow cytometry, *n* = 3. **e** Representative fluorescent images of CD8 makers in tumor slices after different treatments. Scale bars, 50 μm, *n* = 3. All data were analyzed with one-way analysis of variance test. All error bars are expressed as ±SD

We analyzed the levels of cell apoptosis markers in order to understand enhanced antitumor effect of ER-targeting FAL-ICG-HAuNS/FAL-Hb-lipo therapy. Comparing to non-targeting ICG-HAuNS and ICG-HAuNS plus Hb-lipo, FAL-ICG-HAuNS and FAL-ICG-HAuNS plus FAL-Hb-lipo under NIR light treatment caused significant overexpression of the CHOP protein. In addition, we detected cleaved caspase-3 in all the treatment groups (Fig. 6a, b). The data demonstrated that ER-targeting PDT/PTT could significantly cause ER stress and ER-related cell apoptosis and simultaneously induce mitochondria-based cell apoptosis indirectly.

Since ER stress was one of the crucial ICD inducers, the membrane expression of CRT mediated by ER stress was determined. As expected, the ER-located FAL-ICG-HAuNS was able to trigger more CRT exposed on the cell surface compared with the lysosome-located ICG-HAuNS. In addition, thanks to the simultaneously provided oxygen in the ER, FAL-Hb-lipo considerably increased the CRT expression caused by FAL-ICG-HAuNS (Fig. 6c), which could be attributed to the severe ER stress (Fig. 6a).

CRT acts as an “eat me” signal by binding to the surface maker CD91 on DCs, stimulating them to grow into matured DCs, which could present the tumor antigens to T cells for activating subsequent immune responses^26,27^. Therefore, we assessed DC maturation after different treatments by analyzing major histocompatibility complex (MHC) I^+^ and, MHC II^+^ DCs using flow cytometry. MHC I and MHC II are considered as the most important complex in antigen binding inside DCs^28,29^. In lymph nodes, which are the main organs for DC maturation, the MHC I^+^/MHC II^+^ DCs jumped to 61.6% in the FAL compared to 10.9% in the saline group (Fig. 6d). FAL-ICG-HAuNS alone also induced activation in 33.2% DCs. Moreover, FAL-ICG-HAuNS, with the assist of FAL-Hb-lipo, caused 33.1% DC maturation in the tumors due to the massive CRT exposure, a level much higher than that in the other four groups. The results indicated that, compared with non-targeting nanosystems, ER-targeting FAL-ICG-HAuNS plus FAL-Hb-lipo was capable of triggering direct ROS-based ER stress, leading to increased CRT exposure and DC activation under NIR laser irradiation.

Antigen presentation to T cells by matured DCs activates the adaptive immune responses, which is characterized by the formation of cytotoxicity T lymphocyte (CD8^+^ T cells) against cancer cells^30^. As displayed by immunofluorescent staining of spleen tissue slices, amount of CD8^+^ T cells increased in the FAL-ICG-HAuNS plus FAL-Hb-lipo group compared to the other four groups, indicating a stronger immune response after ER-targeting therapy (Supplementary Figs. 9 and 10). More CD8^+^ T cells were recruited into the tumors in the group and CD4^+^ T cells (mostly regulatory T cells (Tregs), as the immune-inhibiting cells) were reduced (Fig. 6e, Supplementary Figs. 10 and 11).

Cytokines secreted by lymphocytes in blood circulation was further detected using the enzyme-linked immunosorbent assay (ELISA). As displayed in Supplementary Fig. 12, tumor necrosis factor (TNF)-α and interferon (IFN)-γ increased in the FAL-ICG-HAuNS alone and FAL-ICG-HAuNS plus FAL-Hb-lipo groups. Their levels showed almost no changes after treatment with ICG-HAuNS, even under the help of Hb-lipo, which was the result of a weak ER stress (Figs. 4a and 6a), little CRT expression (Figs. 4c and 6c), and low level of matured DCs (Fig. 6d). These results illustrated that the ICD caused by ER-targeting PDT/PTT was capable of increasing DC maturation, CD8^+^ T cell proliferation, and cytotoxic cytokine production. The closely linked specific ER stress–ICD (CRT exposure)–DC maturation–immune activation led to the striking antitumor effect caused by ER-targeting FAL-ICG-HAuNS plus FAL-Hb-lipo group under laser mediation (Fig. 5b, c).

### Relief of tumor hypoxia microenvironment

**Fig. 7:**
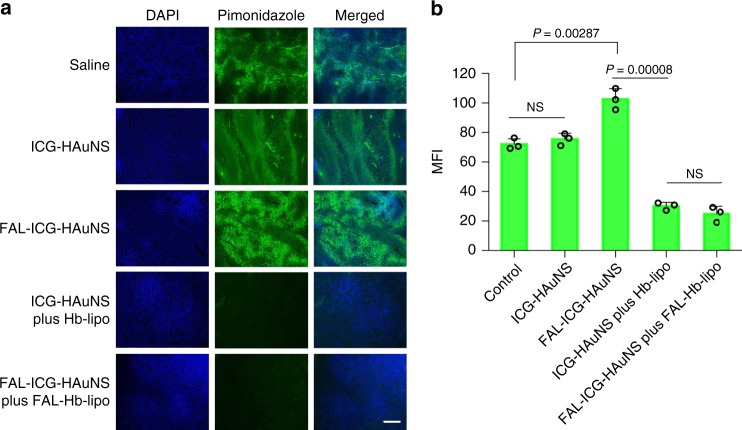
Relief of tumor hypoxia microenvironment in CT-26 model. Laser power: 1 W/cm^2^, 2 min. **a** Representative hypoxia fluorescent photomicrographs of tumors in each group. Scale bars, 200 μm. **b** Mean fluorescence intensity in the tumor slices (*n* = 3). All data were analyzed with one-way analysis of variance test. All error bars are expressed as ±SD. “NS” indicates “not significant”

Since Hb functions as an oxygen carrier, which could release oxygen to a hypoxic microenvironment, it was applied as an adjuvant to relieve the hypoxia status after PDT^18,19^. It was obvious that the hypoxia conditions became worse after FAL-ICG-HAuNS plus laser treatments, which possibly resulted from oxygen consumption of PDT (Fig. 7a, b). In contrast, the hypoxia tumor microenvironment was relieved after the introduction of Hb-lipo or FAL-Hb-lipo. In addition, Hb could supply oxygen to meet the need after repeated PDT (Fig. 5b).

### Antitumor efficacy in B16 tumor model

**Fig. 8:**
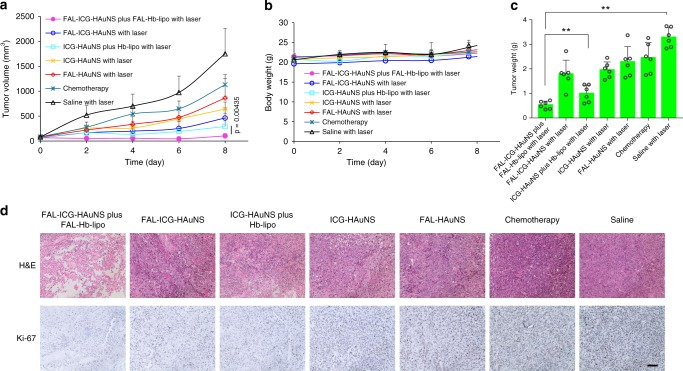
Antitumor efficacy in B16 tumor model. **a** Growth curves of B16 tumors after treatment with saline, chemotherapy (Taxotere: 10 mg/kg per injection), FAL-HAuNS, ICG-HAuNS, FAL-ICG-HAuNS, ICG-HAuNS plus Hb-lipo, or FAL-ICG-HAuNS plus FAL-Hb-lipo (ICG: 0.5 mg/kg, HAuNS: 1 mg/kg, Hb: 20 mg/kg per injection). Taxotere, ICG-HAuNS, or FAL-ICG-HAuNS were injected on days 0, 2, and 4. Hb-lipo and FAL-Hb-lipo were injected on days 1, 3, and 5. Tumors (except the chemotherapy group) were exposed to laser irradiation (1 W/cm^2^, 2 min) on days 1, 3, and 5. *n* = 6. **b** Body weight change curves, *n* = 6. **c** Average tumor weights in different groups at the end of treatment. **d** Representative hematoxylin and eosin and Ki-67 staining photographs in each group. Scale bars, 100 μm. All data were analyzed with one-way analysis of variance test (*P ≤ 0.05; **P ≤ 0.01). All error bars are expressed as ±SD

Comparing to CT-26 tumor, which is relatively more sensitive to multiple therapeutic strategies, B16 is a more resistant tumor model. FAL-HAuNS (PTT alone) and chemotherapy group were added as controls. Antitumor effect was evaluated for 8 days since tumor volume in the saline control group had exceeded 1500 mm^3^ (i.e., meeting the euthanasia endpoint). As shown in Fig. 8a, c, FAL-ICG-HAuNS plus FAL-Hb-lipo group presented significantly stronger antitumor efficacy than the other groups (including non-ER-targeting PTT/PDT, PTT alone, chemotherapy), owing to the specific ROS-based ER stress under sufficient oxygen supply. Histology and proliferation assay revealed the highest cancer cell destruction and lowest proliferative activity in the FAL-ICG-HAuNS plus FAL-Hb-lipo group (Fig. 8d). There was no obvious body weight change or histological lesions in major organs, suggesting that these nanoparticles had a low systemic toxicity profile (Fig. 8b and Supplementary Fig. 13).

### ICD induced by specific ER stress in B16 tumor model

**Fig. 9:**
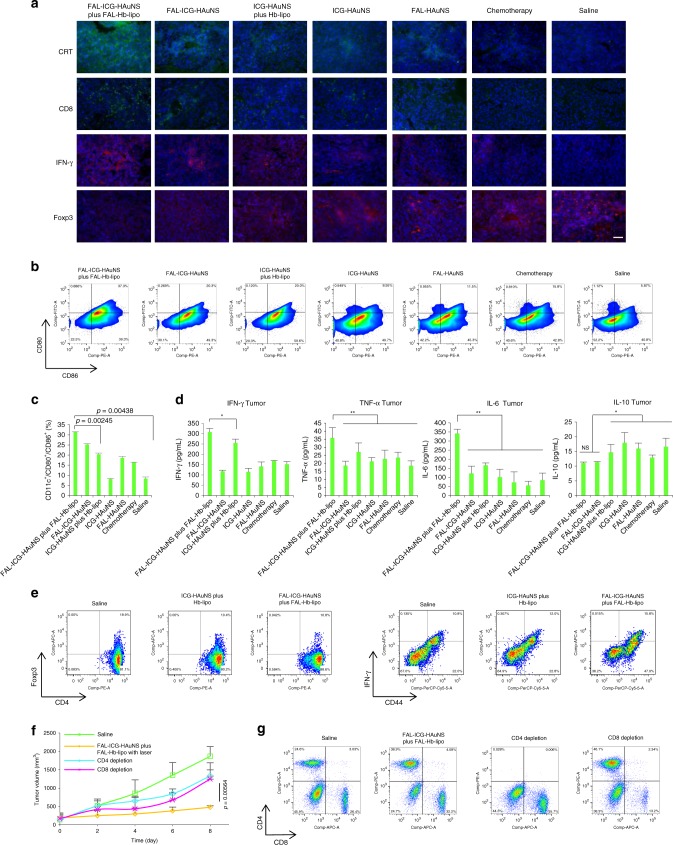
Immunogenic cell death induced by specific endoplasmic reticulum stress in B16 tumor model. **a** Immunofluorescent staining of calreticulin expression, CD8^+^ T cells, interferon (IFN)-γ, or Foxp3^+^ T cells in tumor sections at the end of the treatments. Scale bars, 50 μm. **b** Dendritic cells (DCs) maturation in lymph nodes analyzed with flow cytometry, *n* = 6. **c** Quantification of mature DCs based on the results in **b**, *n* = 6. **d** IFN-γ, tumor necrosis factor-α, interleukin (IL)-6, and IL-10 levels in tumor detected with the enzyme-linked immunosorbent assay, *n* = 6. **e** Representative flow cytometric plots of regulatory T cells (CD3^+^/CD4^+^/Foxp3^+^) and activated CD8^+^ T cells (CD3^+^/CD8^+^/CD44^+^ or CD3^+^/CD8^+^/INF-γ) in the spleens after various treatments. **f** The growth curves of B16 tumors after treatment with saline or FAL-ICG-HAuNS plus FAL-Hb-lipo (ICG: 0.5 mg/kg, HAuNS: 1 mg/kg, Hb: 20 mg/kg per injection). FAL-ICG-HAuNS was injected on days 0, 2, and 4. FAL-Hb-lipo was injected on days 1, 3, and 5. Tumors (except the chemotherapy group) were exposed to laser irradiation (1 W/cm^2^, 2 min) on days 1, 3, and 5. As for CD4 or CD8 depletion group, either CD4^+^ or CD8^+^ T cells were depleted in vivo with anti-CD8 or anti-CD4 antibody (intraperitoneal, 100 μg/mice per injection on days −3, 0, 2, and 4), *n* = 6. **g** Representative flow cytometric plots of CD8^+^ and CD4^+^ T cell populations in the spleen after depletion. All data were analyzed with one-way analysis of variance test (*P ≤ 0.05; **P ≤ 0.01). All error bars are expressed as ±SD

Immune response was further investigated in mice with B16 tumors after multiple drug treatments. As shown in Fig. 9a, comparing to the non-ER-targeting groups, ER-targeting FAL-ICG-HAuNS plus FAL-Hb-lipo showed increased level of ecto-CRT, a marker of ICD^13^. The danger signal acted as an “eat me” signal^13,14,31^, which efficiently promoted DC maturation (CD11c^+^/CD80^+^/CD86^+^) (Fig. 9b, c). Matured DCs could exhibit immunogenic functional stimulation (NO^high^/IL-10^absent^/IL6^high^/IL1β^high^)^13,14^, which was further evaluated. As shown in Fig. 9d, interleukin (IL)-6 in tumors stimulated by matured DCs significantly increased in the FAL-ICG-HAuNS plus FAL-Hb-lipo group. It should be noted that IL-10 obviously decreased in ER-targeting PDT/PTT groups. DC-derived IL-10 is uniquely absent for ER-targeting PDT/PTT-induced ICD but not for chemotherapy-mediated ICD^13,21^.

Mature immunogenic DCs elicited by ER-targeting PDT/PTT-induced ICD further stimulate adaptive immune responses, including CD4^+^ and CD8^+^ T cell proliferation and cytotoxic cytokine (such as TNF-α and IFN-γ) production^13,14,32^. As displayed in Supplementary Fig. 14, CD4^+^ T cells and CD8^+^ T cells in the spleen increased in the FAL-ICG-HAuNS plus FAL-Hb-lipo group. In addition, infiltration of CD3^+^ T cells in tumor tissue elevated to 36.5% (Supplementary Fig. 15). The levels of CD8^+^ T cells, IFN-γ, and Foxp3^+^ cells in tumors were further evaluated with immunofluorescent staining (Fig. 9a). Enhanced fluorescent intensity of CD8^+^ and IFN-γ indicated a strong antitumor immune activity. Furthermore, the amount of immunosuppressive cells such as Treg cells (Foxp3^+^ cells) decreased in the ER-targeting PDT/PTT groups. The treatments induced an obvious increase in cytotoxic cytokine (TNF-α and IFN-γ) levels in tumor tissues (Fig. 9d). In order to investigate populations of Treg cells and activated CD8^+^ cells, CD3^+^/CD4^+^/Foxp3^+^ cells and CD3^+^/CD8^+^/IFN-γ^+^ or CD3^+^/CD8^+^/CD44^+^ cells in the spleens of three representative groups were analyzed with flow cytometry. As shown in Fig. 9e, comparing to the non-ER-targeting group (ICG-HAuNS plus Hb-lipo), the amount of Treg cells (CD3^+^/CD4^+^/Foxp3^+^ cells) in the FAL-ICG-HAuNS plus FAL-Hb-lipo group decreased to about 10%, indicating that the immunosuppressive microenvironment could be improved by ER-targeting PDT/PTT. In addition, the CD3^+^/CD8^+^/IFN-γ^+^ cells and CD3^+^/CD8^+^/CD44^+^ in the FAL-ICG-HAuNS plus FAL-Hb-lipo group were both higher than the other two groups, suggesting activation of CD8^+^ T cells.

In order to investigate the contribution of immune system to the antitumor effect of ER-targeting PDT/PTT, CD4^+^ or CD8^+^ T cells were depleted in vivo with antibodies. Depletion of either CD4^+^ or CD8^+^ T cells abrogated the antitumoral effects of FAL-ICG-HAuNS plus FAL-Hb-lipo. However, compared to the saline group, FAL-ICG-HAuNS plus FAL-Hb-lipo still presented the ability of tumor growth suppression (Fig. 9f). As shown in Fig. 9g, it could be concluded that the CD4^+^ or CD8^+^ T cells were successfully depleted. No obvious body weight change was observed in the whole treatment process (Supplementary Fig. 16). The results indicated that immune system played an important role in the antitumor effect. ICD induced by specific ER stress and the PDT/PTT-mediated cell damage worked together to exhibit a strong tumor inhibition effect.

## Discussion

Induction of ICD presents a therapeutic modality for cancer treatment, which is attributed to its ability of allowing the immune system to eradicate cancer cells through a “bystander effect”^33,34^. Since severe and focused ER stress is essential for ICD, most chemotherapy, radiotherapy, and non-targeting PDT are unable to induce effective ICD owing to secondary or collateral ER stress effects^35,36^. Thus there is a need for an ICD inducer capable of triggering ER stress directly and effectively.

Combined PDT/PTT have been extensively studied by researchers to establish effective nanotherapeutics under light irradiation with a suitable wavelength^37^. Although this strategy showed extremely stronger tumor growth inhibition effect over monotherapy, aggravated tumor hypoxia level after PDT might prevent tumor elimination^16^.

The ER-targeting and hypoxia-reverse nanosystem presented in this study combines FAL peptide-modified PTT/PDT nanosphere (FAL-ICG-HAuNS) with oxygen-delivering liposome (FAL-Hb-lipo). Our previous studies have demonstrated uneven distribution of gold nanospheres in tumors after intravenous (i.v.) injection, which will limit the efficacy from subsequent PDT^38^. Consequently, multiple doses and laser irradiations were performed in our previous^39^ and current studies to reduce heterogeneity of PDT in the tumor. We demonstrated that the double “ER missiles” (FAL-ICG-HAuNS and FAL-Hb-lipo) was prone to accumulate in ER, while the non-targeting ICG-HAuNS and Hb-lipo were transited to the lysosomes after cellular internalization (Fig. 2). FAL-Hb-lipo exhibited the ability to release previously loaded molecular oxygen, which enabled sustained ROS generation in PDT under hypoxia (Fig. 3b). Cytotoxicity from PTT/PDT decreased once oxygen level was low without the oxygen-carrying Hb-lipo (Fig. 3c, d). ER-targeting FAL-ICG-HAuNS showed a stronger cell-killing ability than the non-targeting ICG-HAuNS. Furthermore, cell viability decreased by 16.9% when FAL-ICG-HAuNS was co-administrated with FAL-Hb-lipo compared to that with Hb-lipo at the ICG concentration of 25 μg/mL under hypoxia (Fig. 3c, d).

ER-localized ROS generation mediated by FAL-ICG-HAuNS could cause severe ER stress, as demonstrated by overexpression of the proapoptotic protein CHOP, a marker of ER apoptosis^24^. CHOP level further increased in the presence of FAL-Hb-lipo (Figs. 4a and 6a). Our ER-targeting nanosystem could be used as an effective ICD inducer owing to the specific ROS-based ER stress, which is one of the prerequisite for ICD. Moreover, severe ER stress and ER membrane disruption after PDT might lead to leakage of Ca^2+^, which then induces mitochondria-related cell apoptosis^40,41^. This was demonstrated by the increased level of cleaved caspase-3 (Figs. 4b and 6b). This phenomenon revealed that photodamage of PDT in ER could be spread to other subcellular locations, leading to a stronger antitumor effect.

Various strategies have been developed to address the immune-suppressive microenvironment in malignant tumors, such as with immune checkpoint blockade antibodies targeting PD-1, PD-L1, and CTLA-4^2,42^. In this study, DCs were activated after induction of ICD. ER stress induced activation of the downstream PERK (protein kinase RNA-like ER kinase)^43^. The subsequent phosphorylation of eIF2α (eukaryotic translation initiation factor 2α) was the key for ICD^44^. CRT in ER lumen was transferred to Golgi apparatus, followed by exocytosis of CRT-containing vesicles to the cell surface resulting in ICD induction. As we observed in Figs. 4c–f, 6c, and 9a, CRT level was significantly elevated on the cell membrane after laser-activated ER-targeting nanosystem treatments comparing to non-targeting ones. As shown in Fig. 4g, CRT exposure decreased in the presence of antioxidants, indicating that ROS-based ER-stress was crucial for inducing ICD. The distinct DC maturation and corresponding immunogenic functional stimulation (IL6^high^/IL10^absent^) in both lymph nodes and tumors verified ICD (Figs. 6d and 9b–d).

HAuNS and liposomes were able to specifically deliver drugs to tumor sites through their enhanced penetration and retention effect (Fig. 5a). With the help of FAL-Hb-lipo, FAL-ICG-HAuNS displayed a significant tumor inhibition effect and an improved survival profile in murine models of CT-26 and B16 tumors (Figs. 5b–d and 8a–d). Compared with ICG-HAuNS plus Hb-lipo, the amount of activated CD8^+^ T cells and immune-promoting cytokines (TNF-α and IFN-γ) increased in the FAL-ICG-HAuNS plus FAL-Hb-lipo group (Figs. 6e and 9a–e and Supplementary Fig. 14), suggesting a robust immune response. In the mean time, the amount of Treg cells decreased (Fig. 9a, e), indicating that the immunosuppressive microenvironment was improved by ER-targeting PDT/PTT. This should be attributed to ICD-mediated immunotherapy under a specific ER stress, which could effectively stimulate the hosts’ immune system against cancer. In addition, depletion of CD4 or CD8 T cells abrogated antitumoral effects from FAL-ICG-HAuNS plus FAL-Hb-lipo, which indicated that the immune system played an important role on the antitumor effect (Fig. 9f, g). Furthermore, the tumor hypoxia microenvironment was greatly reversed thanks to the adjuvant oxygen supply (Fig. 7), which could be administered whenever needed.

**Fig. 10:**
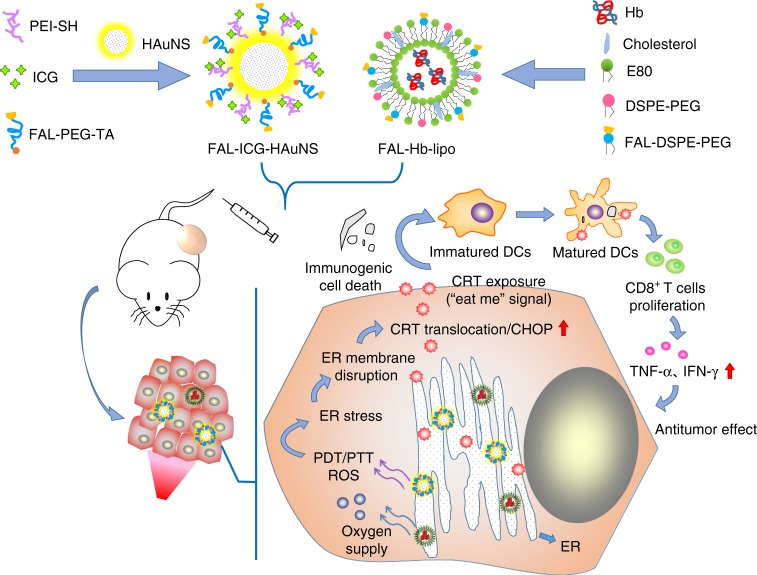
The antitumor mechanism of FAL-ICG-HAuNS plus FAL-Hb-lipo. Schematic illustration of enhanced immunogenic cancer cell death and anticancer effect induced by endoplasmic reticulum-targeting photothermal/photodynamic therapy

Taken together, this study illustrated the concatenation of direct ROS-based ER stress–CRT exposure–DC maturation–immune activation (Fig. 10). The double “ER missiles” (FAL-ICG-HAuNS and FAL-Hb-lipo) proved to be a controllable cancer PTT/PDT/immunotherapy nanosystem mediated by NIR light.

## Methods

### Reagents

Cobalt chloride hexahydrate (99.99%), sodium citrate (>99%), chloroauric acid trihydrate, phycoerythrin-labeled MHC I antibody (catalog # 12–5957–82), and fluorescein isothiocyanate (FITC)-labeled MHC II antibody (catalog # 11–5321–82) were purchased from Thermo Fisher Scientific (Waltham, MA). Taxotere (Docetaxel Injection) was purchased from Sanofi (Paris, FR). Human Hb, sodium borohydride (99%), MTT reagent, 1-ethyl-3-(3-dimethylaminopropyl) carbodiimide (EDC), Trauts reagent (TR), branched polyethylenimine (PEI; molecular weight (MW) = 10 kDa), oxaliplatin, and 4,6-diamidino-2-phenylindole (DAPI) were acquired from Sigma-Aldrich Inc. (St Louis, MO). 4-Dimethylaminopyridine (DMAP), di-tert-butyl dicarbonate ((BOC)_2_O), and TA were obtained from Aladdin Inc. (Shanghai, China). ICG was purchased from Tokyo Chemical Industry (TCI, Tokyo, Japan). SOSG was acquired from Invitrogen Corp. (Carlsbad, CA). Egg phosphatidyl lipid-80 (E80), distearoyl-sn-glycero-3-phosphoethanolamine-N-[maleimide(polyethylene glycol)-2000] (DSPE-PEG_2000_), NH_2_-DSPE-PEG_2000_, and cholesterol were obtained from lipoid GmbH (Ludwigshafen, Germany). β-Actin antibody (catalog # 60008-1-Ig) and CRT antibody (catalog # 10292-1-AP) were supplied by Proteintech Group, Inc. (Wuhan, China). CHOP (catalog # 2895) and caspase-3 (catalog # ab214430) antibodies were obtained from Cell Signaling Technology (Trask Lane, Danvers, MA) and Abcam (Cambridge, MA), respectively. Pimonidazole hydrochloride (hypoxyprobe-1 plus kit) was acquired from Hypoxyprobe (Burlington, MA). FITC-CD3 antibody (catalog # 100203), PE-CD4 antibody (catalog #100407), PE-IFN-γ antibody (catalog # 505807), and APC-CD8α antibody (catalog # 100712) for flow cytometry (fluorescent-activated cell sorting) assay were purchased from Biolegend (California, MA). Anti-rabbit IgG (H+L) (catalog # 70-GAR0072), anti-mouse IgG (H+L) (catalog # 70-GAM007), and APC-Foxp3 antibody (catalog # AM0F05-50) was acquired from MultiSciences (Hangzhou, China). *InVivo*mAb anti-mouse CD8α (clone 53–6.7, catalog # BE0061) and antimouse CD4 (cloneGK1.5, catalog # BE0003-1) were purchased from BioXcell (West Lebanon, NH). BD Cytofix/Cytoperm Kit was acquired from BD Biosciences (New York, MA). ROS detection kit (DCFH-DA), enhanced BCA protein assay kit, and ECL Plus Kit were purchased from Beyotime Institute of Biotechnology (Jiangsu, China). Sodium dodecyl sulfate-polyacrylamide gel electrophoresis (SDS-PAGE) Bis-Tris Gel was acquired from Invitrogen (Carlsbad, CA). FITC-labeled goat-anti-rabbit secondary antibody (catalog # WD-GAR001) and nitrocellulose membrane were from Da Wen Biotechnology Co., Ltd. (Hangzhou, China). Pardaxin (FAL) peptide (H-GFFALIPKIISSPLFKTLLSAVGSALSSSGGQE-OH) was synthesized by Qiang Yao Biotechnology Co. Ltd. (Shanghai, China). NH_2_-PEG_2000_-NH_2_ was acquired from Yare Biotech, Co., Ltd. (Shanghai, China). All the chemicals and solvents were of analytical grade and used as received.

### Cell culture and animal model

CT-26 (mouse colon carcinoma) and B16 (mouse melanoma) cells were cultured in RPMI-1640 medium and Dulbecco’s modified Eagle’s medium, respectively, with 21% O_2_ and 5% CO_2_ at 37 °C. To mimic the hypoxia tumor microenvironment, cells were placed in an incubator with 2% O_2_ and 5% CO_2_ at 37 °C. All culture media contained 10% fetal bovine serum (FBS; Bioind, Cromwell, USA) and 100 U/mL penicillin–streptomycin.

All animal experiments complied with all relevant ethical regulations. The program of the animal experiments in this study (application number: 14413) was approved by Zhejiang University Experimental Animal Welfare Ethics Review Committee. Balb/c male mice (6–8 weeks old) were subcutaneously inoculated with CT-26 cells or B16 cells (10^6^/mice) at the axilla of back legs. Once the tumors reached 50–200 mm^3^ in size, mice were used for further experiments.

### Synthesis of FAL-DSPE-PEG_2000_

FAL-DSPE-PEG_2000_ was obtained after the reaction between NH_2_-DSPE-PEG_2000_ and FAL peptides. First, the amino groups on FAL peptides were protected by (BOC)_2_O. Forty mg FAL were dissolved in 3 mL anhydrous dimethylformamide (DMF) and mixed with (BOC)_2_O at a mol ratio of 1:5.2, followed by the stirring for 12 h under ice bath in dark. Subsequently, EDC and N-Hydroxysuccinimide (NHS) were added into the reaction liquid (FAL:EDC:NHS = 1:5:10, mol ratio) and stirred for 2 h to activate the carboxyl groups on FAL. Then NH_2_-DSPE-PEG_2000_ (FAL:NH_2_-DSPE-PEG_2000_ = 1:1, mol ratio) was added and stirred for 24 h. To remove the protecting groups on FAL, 1 mL HCL (12 M) was further added. Afterwards, the pH value was adjusted to 7.4 by NaOH (3 M). The reaction product was dialyzed with pure water for 48 h and lyophilized to obtain FAL-DSPE-PEG_2000_.

### Synthesis of FAL-PEG_2000_-TA

NH_2_-PEG-TA was synthesized through the dehydration reaction between NH_2_-PEG-NH_2_ and TA. First, TA, Dicyclohexylcarbodiimide (DCC), and NHS (mol ratio 1:5:10) were dissolved in DMF and stirred for 2 h at 60 °C to activate the carboxyl groups on TA. Then certain amount of NH_2_-PEG-NH_2_ was added (NH_2_-PEG-NH_2_:TA = 1:2, mol/mol) and the stirring was continued for another 24 h. The raw product was dialyzed with distilled water for 48 h and then lyophilized to obtain NH_2_-PEG-TA. Before the synthesis of FAL-PEG-TA, the amino groups on FAL peptide were also protected by (BOC)_2_O using the above-mentioned methods. After that, EDC and NHS (FAL:EDC:NHS = 1:5:10, mol ratio) were employed to activate the carboxyl groups on FAL. Then NH_2_-PEG-TA (FAL:NH_2_-PEG-TA = 1:1, mol/mol) was added and the stirring was continued for another 24 h. At the end of the reaction, HCI were used for removing the protecting groups and the pH value was titrated to 7.4 by NaOH. After further dialysis and lyophilization, FAL-PEG-TA was collected and stored at 4 °C before use.

### Preparation of Hb-lipo and FAL-Hb-lipo

To prepare liposomes containing Hb (Hb-lipo), 4.92 mg E80, 1.25 mg cholesterol, and 0.72 mg DSPE-PEG_2000_ were dissolved in 5 mL chloroform and then evaporated at 45 °C using a rotary evaporator to form a thin film (to prepare FAL-modified Hb-lipo (FAL-Hb-lipo), 0.72 mg DSPE-PEG_2000_ were replaced by 0.54 mg DSPE-PEG_2000_ and 0.1 mg FAL-DSPE-PEG_2000_). Afterwards, Hb solution (5 mg/mL in PBS, pH = 7.4) was added into the flask, which was oscillated for 10 min to hydrate the lipid film. Hb-lipo or FAL-Hb-lipo was obtained after further sonication (40 W, 8 min) under ice bath.

Particle size of liposomes was measured using a Zetasizer (Nano S90, Malvern, UK). Particle morphology was observed with TEM (JEM-1230, JEOL, Japan) and absorption spectrum was recorded with a UV-vis spectrophotometer (Agilent Cary 60 UV-vis, Santa Clara, CA). Drug content was determined with a spectrophotometer after demulsification.

Hb-lipo and FAL-Hb-lipo solutions were replenished with O_2_ for 15 min. Oxygen content in Hb-lipo or FAL-Hb-lipo was detected using a dissolved oxygen meter (JPBJ-607A, Rex, China).

### Synthesis of ICG-HAuNS and FAL-ICG-HAuNS

HAuNS were first synthesized. Briefly, 2.8 mL sodium citrate (0.294 g/mL) were added into pre-deoxygenated water, followed by the blend of 4 mL sodium borohydride (0.04 g/mL) and 1 mL cobalt chloride (0.095 g/mL) under intense stir to form homogeneous cobalt nanospheres. Chloroauric acid (0.04 g/mL) was then added so as to be reduced on the surface of the nanospheres. The reaction solution was then exposed to air, resulting in the oxidation of the internal cobalt nanospheres. HAuNS were purified by repeatedly centrifuging the final solution at 10,000 rpm for 10 min.

The surface of HAuNS were modified with thiolated PEI (MW = 10 kDa) and then conjugated with ICG. Specifically, TR was mixed with PEI (PEI:TR = 1:5, mol ratio, pH = 7–10) under continuous stir for 20 min. Thiolated PEI (PEI-SH) was obtained by transforming parts of its primary amino groups into hydrosulfide groups. Then PEI-SH was added into DMF solution containing HAuNS and stirred for another 6 h. PEI-HAuNS were collected after removing the unreacted PEI-SH by repeatedly centrifuging (10,000 rpm, 10 min). In all, 0.8 mg ICG was activated by EDC/DMAP (ICG:EDC:DMAP = 1:3:3, mol ratio) at 45 °C and then mixed with PEI-HAuNS (1 mg HAuNS) in deionized water. The dehydration reaction was continued for 24 h. After three times of centrifugation for removing free ICG in the supernatant, ICG-HAuNS were obtained.

To further conjugate FAL peptide, 0.1 mL FAL-PEG_2000_-TA (10 mg/mL) was mixed with 1 mL ICG-HAuNS (ICG: 0.5 mg/mL; HAuNS: 1 mg/mL) and stirred for 24 h. The purification of FAL-ICG-HAuNS was realized by centrifugation (10,000 rpm, 10 min).

Particle size, structure, and absorption spectrum of ICG-HAuNS or FAL-ICG-HAuNS were also determined. The encapsulation efficiency was calculated indirectly by comparing the amount of ICG in the supernatant to that in the total reaction solution.

### Cell internalization and subcellular localization

CT-26 cells were incubated with DiD-labeled Hb-lipo (DiD@Hb-lipo, Hb: 50 μg/mL), DiD-labeled FAL-Hb-lipo (DiD@FAL-Hb-lipo, Hb: 50 μg/mL), ICG-HAuNS (ICG: 25 μg/mL; HAuNS: 50 μg/mL), or FAL-ICG-HAuNS (ICG: 25 μg/mL; HAuNS: 50 μg/mL) for 2, 12, or 24 h. The cell nuclei were stained with DAPI for 15 min and fixed with 4% paraformaldehyde, followed by the observation using a confocal microscopy (A1R, Nikon, Japan. Objectives ×10, Resolution 120 nm, Magnification ×60).

To investigate subcellular localization of the particles, cells were first incubated with DiD@Hb-lipo (Hb: 50 μg/mL), DiD@FAL-Hb-lipo (Hb: 50 μg/mL), ICG-HAuNS (ICG: 25 μg/mL; HAuNS: 50 μg/mL), or FAL-ICG-HAuNS (ICG: 25 μg/mL; HAuNS: 50 μg/mL) for 6 h, followed by staining of ER or lysosomes with ER or Lyso tracker, respectively. Co-localization of nanospheres and organelles was determined using a confocal microscopy (For ICG, Ex:735 nm; for DiD, Ex: 649 nm).

### Intracellular stability

In order to evaluate stability of ICG-HAuNS or FAL-ICG-HAuNS in cancer cells, ICG-HAuNS (ICG: 25 μg/mL; HAuNS: 50 μg/mL) or FAL-ICG-HAuNS (ICG: 25 μg/mL; HAuNS: 50 μg/mL) were incubated with CT-26 cells for 6 h respectively. Cells were washed with PBS for three times and grown in fresh culture medium. Amount of ICG in supernatant at the 2, 12, and 24 h time points was determined using a UV-Vis spectrophotometer. Cells were then lysed, and cell lysate was collected and smashed for determination of ICG concentration. A BCA Protein Assay Kit was used to quantify protein concentration in cell suspension. ICG content in cells at different time point serves as an indicator for intracellular stability and was calculated as *C*_int_/(*C*_con_ − *C*_med_) × 100%. In this formula, *C*_con_ is ICG concentration at 0 h and *C*_int_ and C_med_ are ICG concentrations inside the cells and in medium, respectively.

### Antitumor effect in vitro

In vitro cytotoxicity under normal or hypoxia conditions was measured with the MTT assay. CT-26 cells were incubated with ICG-HAuNS, FAL-ICG-HAuNS, Hb-lipo, FAL-Hb-lipo, ICG-HAuNS plus Hb-lipo, FAL-ICG-HAuNS plus Hb-lipo, or FAL-ICG-HAuNS plus FAL-Hb-lipo at various concentrations (ICG: 0.5, 5, or 25 μg/mL, HAuNS or Hb: 1, 10, or 50 μg/mL) under normal or hypoxia conditions, respectively. Cells were exposed to NIR light irradiation (1 W/cm^2^, 1 min) 4 h later. Cells with no treatment were used as controls. After 48 h incubation, cell viability (survival rate) was measured following the standard method of MTT.

### ROS generation in vitro

Singlet oxygen (^1^O_2_) production was determined under normal and hypoxia conditions, respectively. Free ICG (10 μg/mL), HAuNS (20 μg/mL), ICG-HAuNS (ICG: 10 μg/mL; HAuNS: 20 μg/mL), or ICG-HAuNS (ICG: 10 μg/mL; HAuNS: 20 μg/mL) plus Hb-lipo (20 μg/mL) was mixed with a fluorescent ^1^O_2_ probe, SOSG (1.0 μM). The suspension was treated with laser irradiation (1 W/cm^2^, 10 min) at 808 nm. After centrifugation at 18,000 rpm for 10 min, supernatant was collected for detection of fluorescence (Ex: 498 nm, Em: 525 nm) using a spectrophotometer (F-2500, HITACHI, Co., Japan). Suspensions protected from light exposure were used as controls. The hypoxia environment was achieved by N_2_ saturation.

Intracellular ROS generation was determined by the ROS detection kit (DCFH-DA as a probe). CT-26 cells were incubated for 6 h with free ICG (10 μg/mL), ICG-HAuNS (ICG: 10 μg/mL; HAuNS: 20 μg/mL), FAL-ICG-HAuNS (ICG: 10 μg/mL; HAuNS: 20 μg/mL), ICG-HAuNS plus Hb-lipo (ICG: 10 μg/mL; HAuNS: 20 μg/mL; Hb: 20 μg/mL), or FAL-ICG-HAuNS plus FAL-Hb-lipo (ICG: 10 μg/mL; HAuNS: 20 μg/mL; Hb: 20 μg/mL). Cells were then exposed to 808 nm laser irradiation at the power dose of 1 W/cm^2^ for 2 min. After immediate incubation with DCFH-DA (10 μM/L) for 20 min, cells were examined with a fluorescence microscopy (Nikon Eclipse, Nikon, Japan; pinhole size 1.49, Objectives ×10, Magnification ×40; Ex: 488 nm) to determine ROS level. ROS up (50 μg/mL) was used as the positive control, which could stimulate intracellular ROS production. Mean intracellular fluorescence intensity was analyzed with the “ImageJ” software.

### Cell apoptosis mechanism study

CT-26 cells were first incubated for 12 h with free ICG (20 μg/mL), ICG-HAuNS (ICG: 20 μg/mL; HAuNS: 40 μg/mL), FAL-ICG-HAuNS (ICG: 20 μg/mL; HAuNS: 40 μg/mL), ICG-HAuNS (ICG: 20 μg/mL; HAuNS: 40 μg/mL) plus Hb-lipo (40 μg/mL), or FAL-ICG-HAuNS (ICG: 20 μg/mL; HAuNS: 40 μg/mL) plus FAL-Hb-lipo (40 μg/mL). Cells were then irradiated with 808 nm laser (2 W/cm^2^, 2 min). They were rinsed with PBS 3 h later and then lysed with CelLytic M cell lysis buffer containing a protease inhibitor cocktail. Cell lysate was centrifuged at 10,000 rpm for 10 min and protein content in supernatant was measured using an enhanced BCA Protein Assay Kit. Equal amounts of protein were electrophoresed in an SDS-PAGE Bis-Tris Gel and then transferred onto nitrocellulose membranes. Membranes were blocked with 5% nonfat dry milk for 2 h at room temperature and then incubated overnight at 4 °C with caspase-3 (dilution 1:5000) or CHOP (dilution 1:1000) antibody, followed by incubation with a horseradish peroxidase-conjugated secondary antibody (dilution 1:2000) for 1 h. Labeled proteins were visualized using the ECL Plus Kit. Uncropped scans for blots are presented in Supplementary Fig. 17.

### Measurement of cell surface CRT

Overexpression of CRT was investigated using immunofluorescent staining. CT-26 cells were first incubated for 12 h with oxaliplatin (40 μM, a positive control for ER stress with CRT expression), ICG-HAuNS (ICG: 10 μg/mL; HAuNS: 20 μg/mL), FAL-ICG-HAuNS (ICG: 10 μg/mL; HAuNS: 20 μg/mL), ICG-HAuNS (ICG: 10 μg/mL; HAuNS: 20 μg/mL) plus Hb-lipo (50 μg/mL), or FAL-ICG-HAuNS (ICG: 10 μg/mL; HAuNS: 20 μg/mL) plus FAL-Hb-lipo (50 μg/mL). VE (150 μg/mL) was used as a reducing agent to prevent ROS production. Cells were irradiated with 808 nm laser (1 or 3 W/cm^2^, 2 min) and cultured for another 24 h. They were then rinsed with PBST, fixed with 4% formaldehyde, and incubated with 5% FBS for 30 min at 37 °C. Rabbit anti-CRT antibody (dilution 1:100) was added and incubated for 12 h at 4 °C. After rinsing with PBST for three times, cells were stained with FITC-labeled goat-anti-rabbit secondary antibody (dilution 1:150) and nuclei were stained with DAPI. CRT expression was measured using a fluorescence microscope.

### Measurement of total CRT protein

CT-26 cells were first incubated with ICG-HAuNS (ICG: 10 μg/mL; HAuNS: 20 μg/mL) or FAL-ICG-HAuNS (ICG: 10 μg/mL; HAuNS: 20 μg/mL). They were irradiated 12 h later with 808 nm laser (1 or 3 W/cm^2^) for 2 min and then cultured for another 24 h. Cells were rinsed with PBS and then lysed with CelLytic M cell lysis buffer containing a protease inhibitor cocktail. Cell lysate was centrifuged at 10,000 rpm for 10 min and protein content in supernatant was measured using an enhanced BCA Protein Assay Kit. CRT (antibody dilution, 1:1000) protein level was determined by western blot described above. Uncropped scans for blots are presented in Supplementary Fig. 17.

### Biodistribution

To verify the potential of Hb-lipo and FAL-Hb-lipo to act as adjuvant treatments in PDT, biodistribution was investigated with subcutaneous CT-26 tumor models.

Mice bearing 100 mm^3^ tumors were i.v. injected with ICG-HAuNS (ICG: 4 mg/kg; HAuNS: 8 mg/kg), FAL-ICG-HAuNS (ICG: 4 mg/kg; HAuNS: 8 mg/kg), DiD@Hb-lipo (Hb: 20 mg/kg), or DiD@FAL-Hb-lipo (Hb: 20 mg/kg). The mice were imaged with an in vivo imaging system (MK50101-EX, CRI Inc., Woburn, MA) at 2, 6, 12, 24, and 24 h, respectively (for ICG, Ex: 735 nm; for DiD, Ex: 649 nm). They were sacrificed 48 h postinjection and the major organs were imaged.

### Flow cytometric assay—mouse

Mice were sacrificed, and the tumors, spleens, and lymph nodes were isolated and minced using surgical scissors. Tissues were digested with Collagenase II and then passed through a 40-mm filter. After three rounds of PBS washes, single-cell suspensions were harvested and then subjected to fluorescein-conjugated staining. For intracellular staining (such as IFN-γ and Foxp3), samples were incubated with the penetration buffer in BD Cytofix/Cytoperm Kit followed by incubation with antibodies according to the manufacturer’s protocols. The preliminary FSC/SSC gates of the starting cell population were set based on the size of lymphocytes. All samples were subject to flow cytometry (BD Fortessa, Becton Dickinson Company, MA) and analyzed with the FlowJo software.

### Antitumor effect and immune responses in CT-26 tumor model

Balb/c mice bearing 50 mm^3^ CT-26 tumors were randomly divided into five groups (*n* = 6). The mice in groups 1–3 were i.v. injected with saline, ICG-HAuNS (ICG: 0.5 mg/kg; HAuNS: 1 mg/kg), or FAL-ICG-HAuNS (ICG: 0.5 mg/kg; HAuNS: 1 mg/kg) on days 0, 2, and 4. As for the ICG-HAuNS plus Hb-lipo (group 4) and FAL-ICG-HAuNS plus FAL-Hb-lipo (group 5) treatment groups, ICG-HAuNS (ICG: 0.5 mg/kg; HAuNS: 1 mg/kg) or FAL-ICG-HAuNS (ICG: 0.5 mg/kg; HAuNS: 1 mg/kg) were injected on days 0, 2, and 4, and Hb-lipo or FAL-Hb-lipo (Hb: 20 mg/kg) was injected on days 1, 3, and 5. Tumors in each group were exposed to laser irradiation (1 W/cm^2^, 2 min) on days 1, 3, and 5 (6 h after Hb-lipo or FAL-Hb-lipo injection). Tumor size (calculated as length × width^2^/2) and body weight were monitored every other day. On day 20, all mice were sacrificed and H&E staining was performed to analyze tissue damage after various treatments. In addition, Ki-67 staining was carried out to verify cancer cell proliferation. Tumors were grinded, lysed in the presence of RIPA (containing 1 mM PMSF) for 0.5 h on ice, and then passed through a 40-mm filter. The samples were centrifuged (10,000 rpm/10 min) and supernatants were used for analysis of caspase-3 and CHOP protein expression by western blot. Uncropped scans for blots are presented in Supplementary Fig. 17. Based on the administration schedule of in vivo antitumor effect, long-term survival was recorded for up to 2 months in a separate cohort of mice. Mice were euthanized when they reached one of the endpoints: loss of 20% body weight, tumor size >1000 mm^3^, hunched back, or lethargic. In order to evaluate immune responses after the treatments, representative mice were sacrificed on day 20 and their blood samples were collected for cytokine detection (TNF-α and IFN-γ) with ELISA kits according to the manufacturer’s suggestion. Moreover, CD4^+^ and CD8^+^ cells in the spleens or tumors of mice were detected by immunofluorescence staining.

In a separate experiment to investigate activation and maturation of DCs, mice bearing 50 mm^3^ CT-26 tumors were randomly divided into five groups and received treatments as described above. Saline and nanoparticles were injected on day 0, and Hb-lipo and FAL-Hb-lipo were injected on day 1. All tumors were exposed to laser irradiation on day 1. Tumors and lymph nodes were collected 1 week postinjection, and MHC I^+^ and MHC II^+^ DCs were analyzed using flow cytometry.

### Examination of hypoxia status in CT-26 tumor model

Hypoxia status in tumors was examined with immunostaining. At the end of the in vivo antitumor study with CT-26 tumors, pimonidazole hydrochloride (60 mg/kg) was i.v. injected into the mice. Mice were sacrificed 30 min later, and tumors were collected and tissue slides were processed. The samples were immunofluorescence-stained with an antibody (FITC-labeled Hypoxyprobe 1-Mab1, dilution 1:200) for pimonidazole adducts. Hypoxia status in tumor microenvironment after various treatments was determined based on fluorescence from pimonidazole (Ex: 488 nm).

### Antitumor effect and immune responses in B16 tumor model

Mice bearing 100 mm^3^ B16 tumors were randomly divided into seven groups (*n* = 6) and treated (i.v.) with saline, chemotherapy (Taxotere, injected on days 0, 2, and 4; 10 mg/kg per injection), FAL-HAuNS, ICG-HAuNS, FAL-ICG-HAuNS, ICG-HAuNS plus Hb-lipo, or FAL-ICG-HAuNS plus FAL-Hb-lipo (ICG: 0.5 mg/kg, HAuNS: 1 mg/kg per injection) on days 0, 2, and 4. Hb-lipo or FAL-Hb-lipo (Hb: 20 mg/kg) was injected on days 1, 3, and 5. Tumors (except the chemotherapy group) were exposed to laser irradiation (1 W/cm^2^, 2 min) on days 1, 3, and 5 (6 h after Hb-lipo or FAL-Hb-lipo injection). Mice were sacrificed 10 days after the initial injection. Tumor size and body weight were measured every other day. At the end of the experiment, tumors and major organs were excised and embedded in paraffin for H&E and Ki-67 staining.

To examine immune responses after treatments, CRT expression, CD8^+^ T cell population, IFN-γ, and Treg cell (Foxp3^+^) levels in tumor regions were analyzed using immunofluorescence staining. In addition, CD3^+^/CD4^+^ or CD3^+^/CD8^+^ T cells in the spleens and CD3^+^ T cells in tumors were analyzed using flow cytometry. Tregs were detected based on CD3^+^/CD4^+^/Foxp3^+^. CD3^+^/CD8^+^/IFN-γ^+^ or CD3^+^/CD8^+^/CD44^+^ T cells were analyzed for activated CD8^+^ T cells. For detection of DC maturation, CD11c^+^/CD86^+^/CD80^+^ DCs in the spleen were analyzed using flow cytometry. IL-6, TNF-α, IL-10, and IFN-γ in tumors were further examined with ELISA kits.

In a separate experiment to investigate the contribution of immune system to antitumor effect, mice bearing 150 mm^3^ B16 tumors were randomly divided into four groups (*n* = 6) and treated (i.v.) with saline or FAL-ICG-HAuNS plus FAL-Hb-lipo following the administration scheme of antitumor effect. To deplete CD4 or CD8 T cells, after treatment with FAL-ICG-HAuNS plus FAL-Hb-lipo group followed by laser irradiation, mice were treated with intraperitoneal injection of anti-CD4 or anti-CD8 antibody (100 μg/mice per injection on days −3, 0, 2, and 4). Mice were sacrificed 10 days after the initial injection. Tumor size and body weight were measured every other day. At the end of the treatment, CD8^+^ and CD4^+^ T cells in splenic lymphocytes were analyzed with flow cytometry.

### Semi-quantification by ImageJ

For each experiment, three or more fields of view were taken as fluorescent images for each group. Fluorescent intensity in each image was semi-quantitated with ImageJ and averaged. Results were displayed as mean fluorescence intensity.

### Statistical analysis

All data are displayed as representative or results from multiple independent experiments. Data comparisons were performed with one-way analysis of variance test. *P < 0.05 was regarded as statistical significance. **P < 0.01 was considered as extreme statistical significance. All error bars are expressed as ±SD.

### Reporting summary

Further information on research design is available in the Nature Research Reporting Summary linked to this article.

## Supplementary information

Supplementary Information

Reporting Summary

## Data Availability

All data generated or analyzed during this study are included in this published article and the Supplementary information or available from the author upon reasonable request.
