# Supplementary material for: Targeting photodynamic and photothermal therapy to the endoplasmic reticulum enhances immunogenic cancer cell death
Source: Nat Commun. 2019 Jul 26;10:3349. doi: 10.1038/s41467-019-11269-8 (PMC6659660; doi:10.1038/s41467-019-11269-8)
Supplement: Supplementary file 1 — Supplementary Information [file 41467_2019_11269_MOESM1_ESM.pdf]

Supplementary information for

Targeting photodynamic and photothermal therapy to the endoplasmic reticulum enhances immunogenic cancer cell death

Wei Li[# 1], Jie Yang[# 1], Lihua Luo[# 1], Mengshi Jiang[1], Bing Qin[1], Hang Yin[1], Chunqi Zhu[1], Xiaoling Yuan[1], Junlei Zhang[1], Zhenyu Luo[1], Yongzhong Du[1], Qingpo Li[1], Yan Lou[2], Yunqing Qiu[2], Jian You[* 1]

1. College of Pharmaceutical Sciences, Zhejiang University, 866 Yuhangtang Road, Hangzhou, Zhejiang 310058, P. R. China
2. The First Affiliated Hospital of Medical School of Zhejiang University, 79 Qingchun Road, Hangzhou, Zhejiang 310058, P. R. China

* Corresponding Author:
Jian You, College of Pharmaceutical Sciences, Zhejiang University, Yuhangtang Road 866, Hangzhou 310058, People's Republic of China; Tel: 086-571-88981651; Fax: 086-571-88208439; E-mail: youjiandoc@zju.edu.cn.

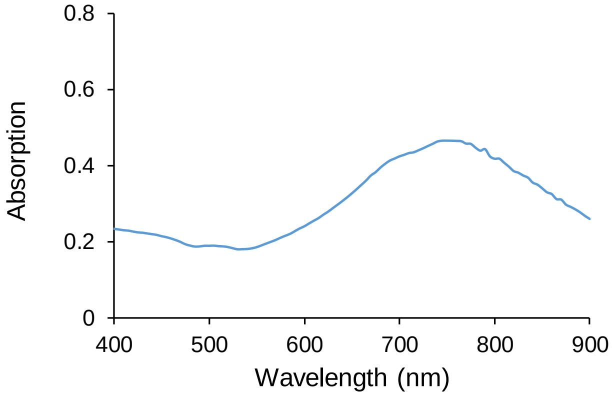

Supplementary Figure 1. UV-vis absorption spectra of HAuNS.

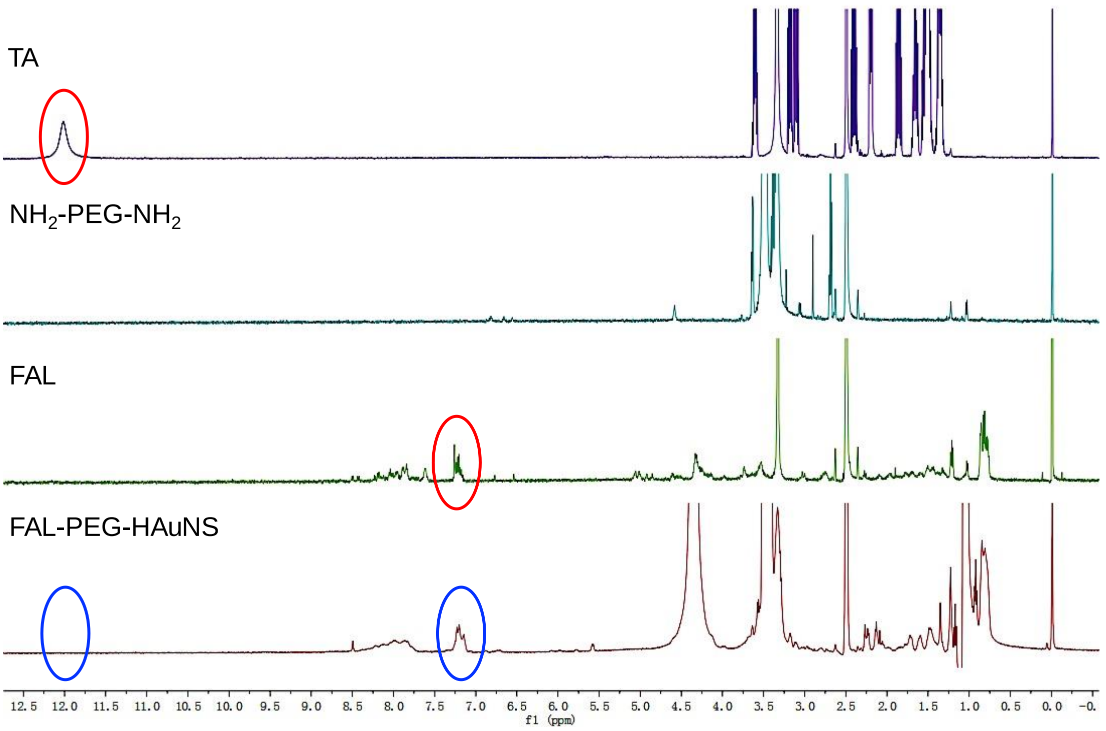

Supplementary Figure 2. ^{1}H NMR spectra of TA, NH_2-PEG-NH_2, FAL and FAL-PEG-HAuNS.

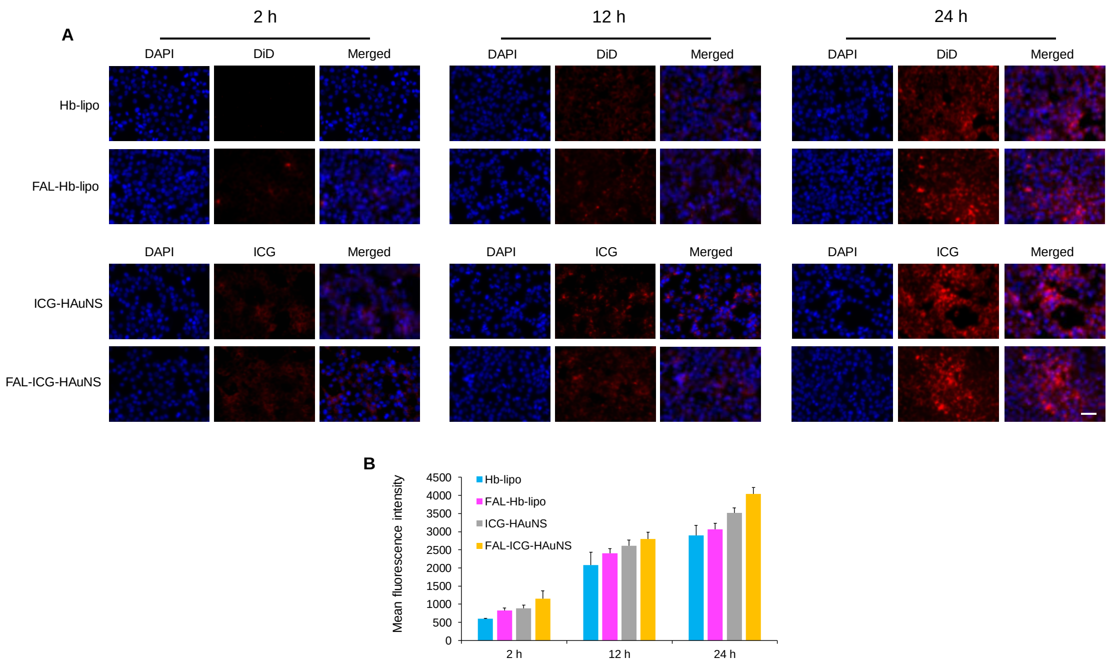

Supplementary Figure 3. Cell internalization. (A) Representative fluorescence images of Hb-lipo, FAL-Hb-lipo, ICG-HAuNS or FAL-ICG-HAuNS in CT-26 cells at 2, 12, 24 h. Scale bars, 50 μm. (B) Fluorescent quantitative analysis of the cell internalization using “Image J” software, n = 3. All error bars are expressed as ± SD.

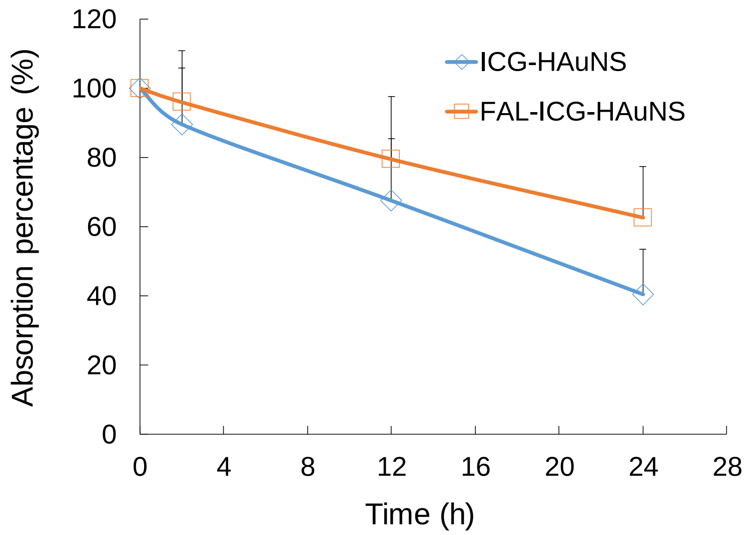

Supplementary Figure 4. Intracellular stability. The intracellular stability of ICG-HAuNS or FAL-ICG-HAuNS with 24 h, n = 3. All error bars are expressed

as ± SD.

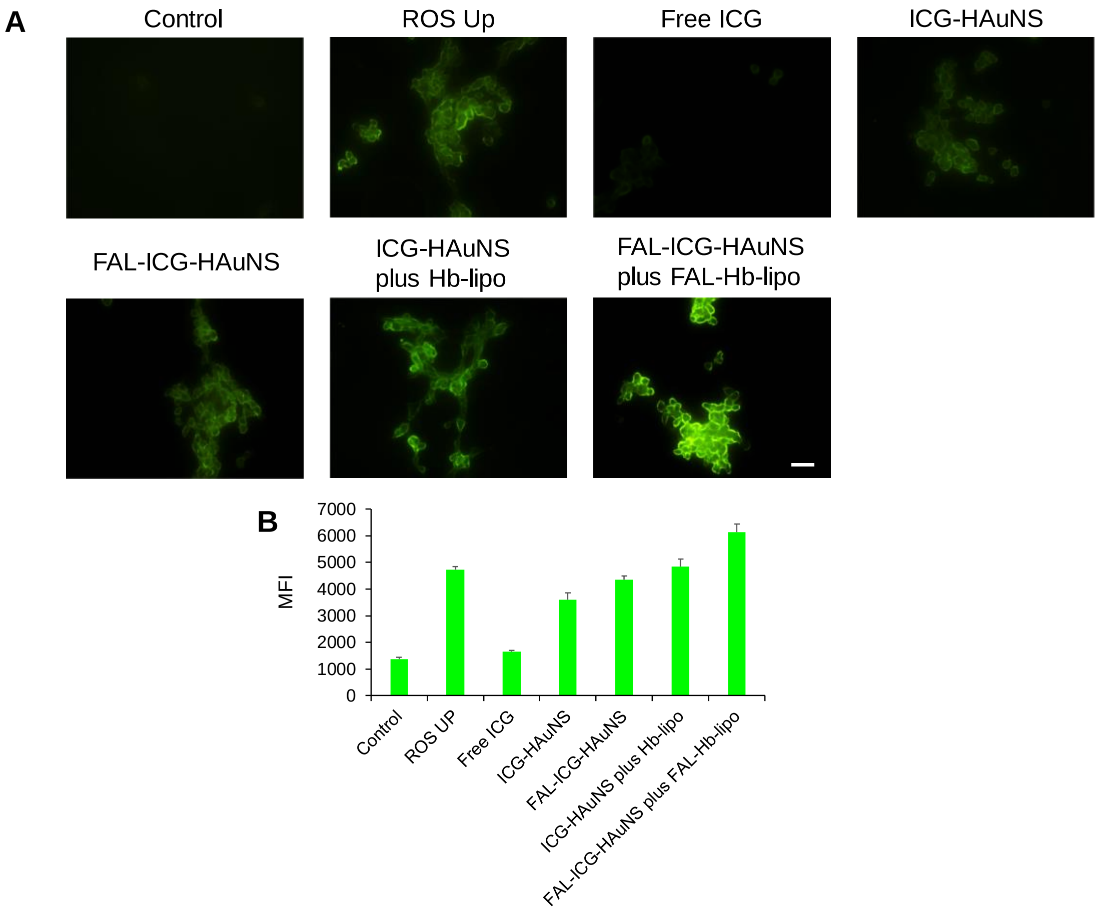

Supplementary Figure 5. Intracellular ROS generation. (A) Intracellular ROS generation of free ICG (10 μg/mL), ICG-HAuNS (ICG: 10 μg/mL; HAuNS: 20 μg/mL), FAL-ICG-HAuNS (ICG: 10 μg/mL; HAuNS: 20 μg/mL), ICG-HAuNS plus Hb-lipo (ICG: 10 μg/mL; HAuNS: 20 μg/mL; Hb: 20 μg/mL), or FAL-ICG-HAuNS plus FAL-Hb-lipo (ICG: 10 μg/mL; HAuNS: 20 μg/mL; Hb: 20 μg/mL) detected by DCFH-DA. Laser power: 1 W/cm^2, 2 min. Scale bars, 20 μm. (B) Fluorescent quantitative analysis of the ROS level using “Image J” software, n = 3. All error bars are expressed as ± SD.

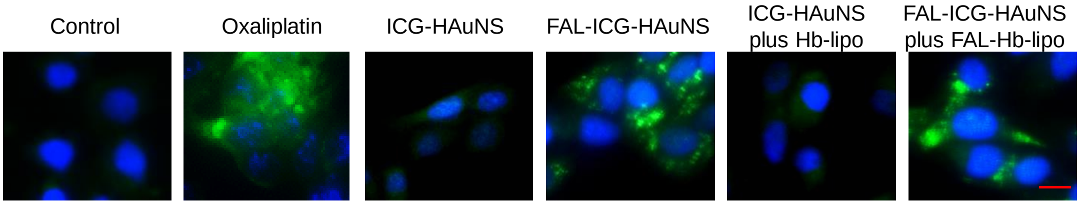

Supplementary Figure 6. Corresponding partial amplification of Figure 4G. Representative fluorescence imaging of CRT exposure with (1 W/cm^2, 2 min) laser irradiation. Oxaliplatin was employed as a positive control for ER stress with CRT expression. Ex: 488 nm. Scale bars, 10 μm, n = 3.

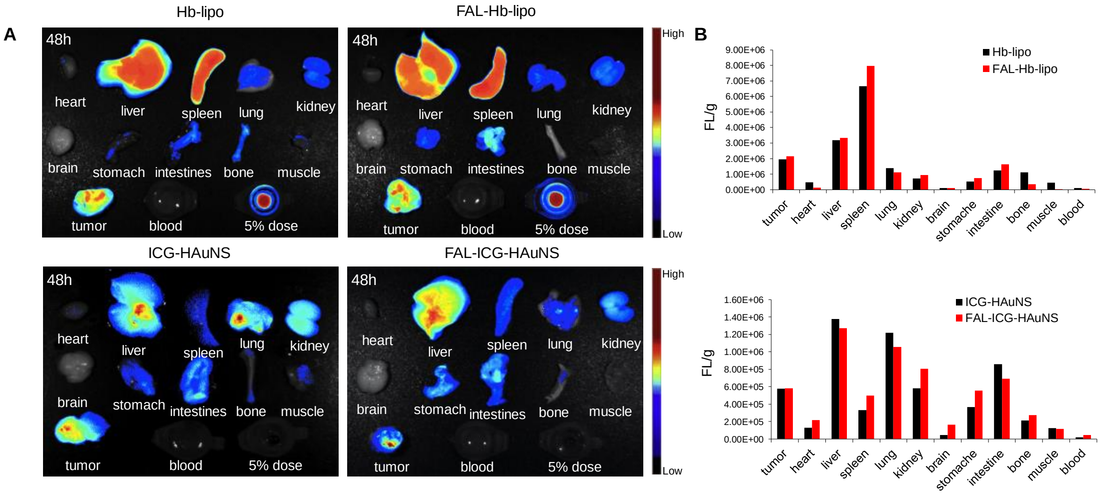

Supplementary Figure 7. Biodistribution. (A) *Ex vivo* fluorescence imaging of the tumors and major organs at 48 h post-injection of Hb-lipo, FAL-Hb-lipo, ICG-HAuNS or FAL-ICG-HAuNS. (B) Semi-quantitative determination of the biodistribution in different organs.

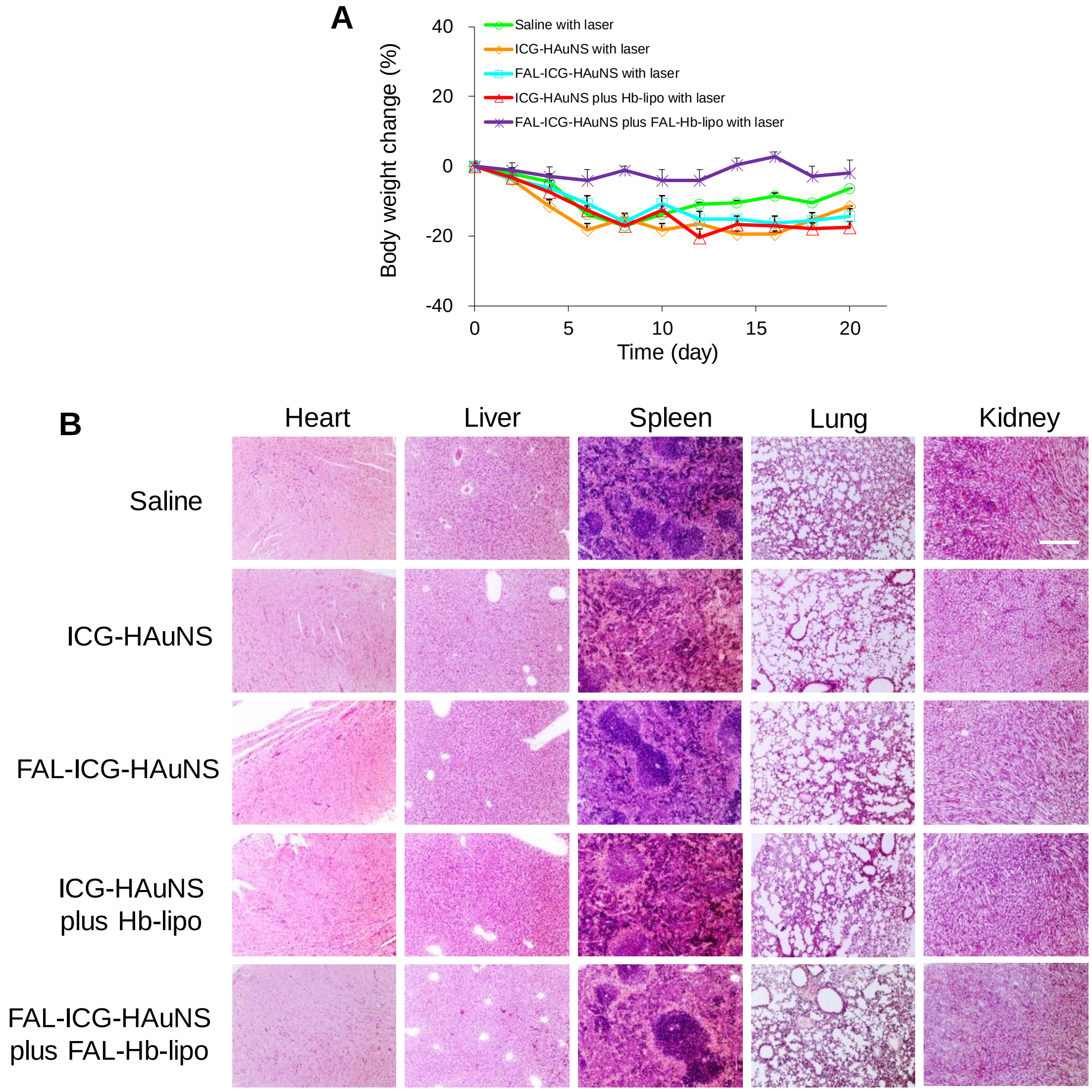

Supplementary Figure 8. Safety study of CT-26 tumor model. (A) Body weight change curves of mice within 20 days, n = 6. (B) Representative H&E staining photographs of heart, liver, spleen, lung and kidney in each group. Scale bars, 100 μm. All error bars are expressed as ± SD.

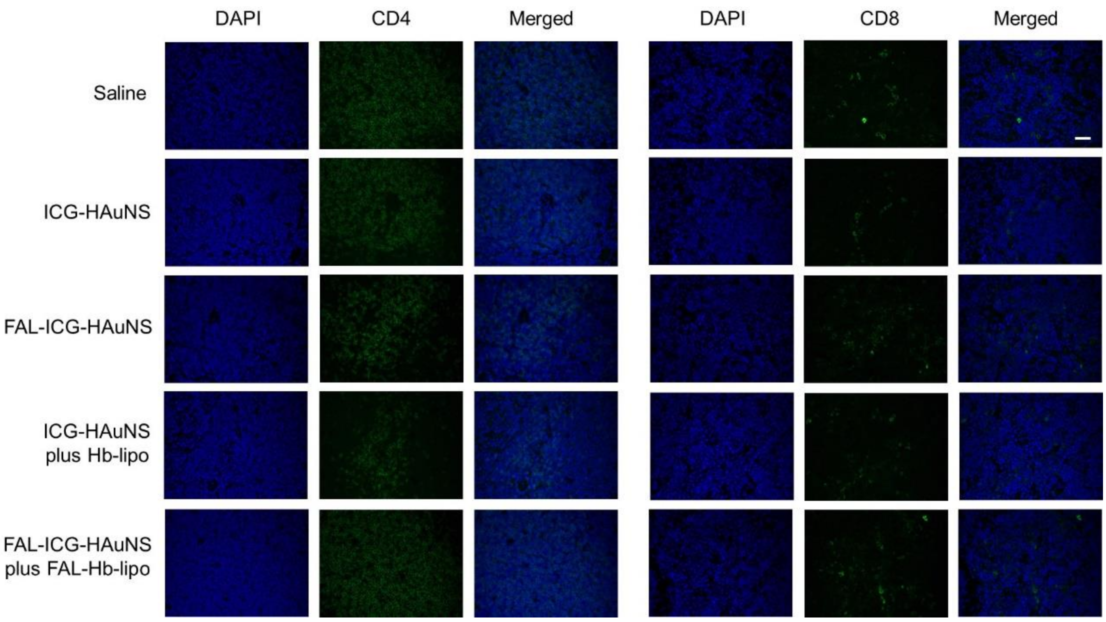

Supplementary Figure 9. Immune responses in spleens. The CD4 and CD8 makers in spleen slices after different treatments analyzed by immunofluorescence staining. Scale bars, 50 μm.

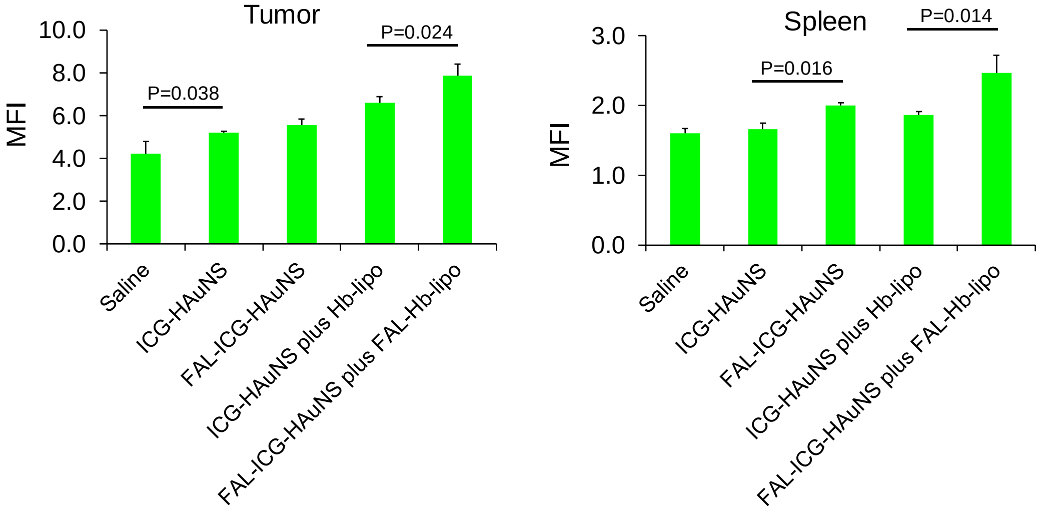

Supplementary Figure 10. Semi-quantification of CD8 makers. The semi-quantification of CD8 makers in tumor and spleen slices after different treatments in Figure 6E and Figure S9, n = 3. All data were analyzed with one-way ANOVA test. All error bars are expressed as ± SD.

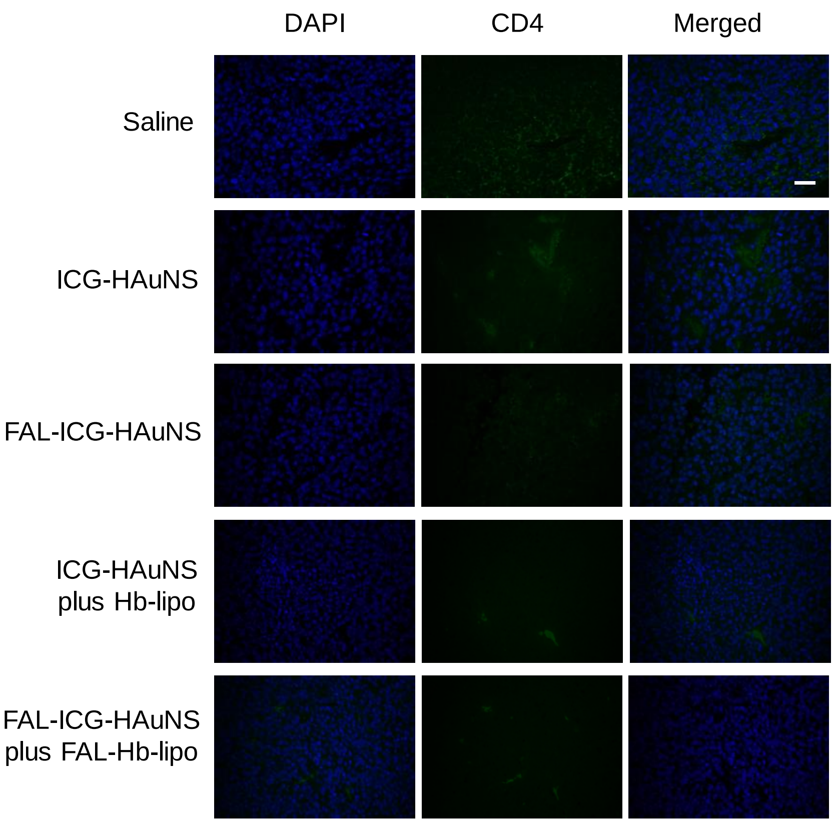

Supplementary Figure 11. Immune responses in tumors. The CD4 makers in tumor slices after different treatments analyzed by immunofluorescence staining. Scale bars, 50 μm.

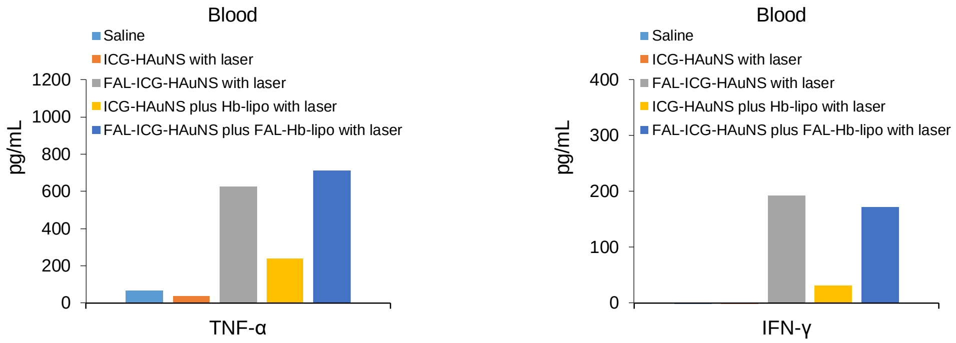

Supplementary Figure 12. Detection of Cytokines. The amount of TNF-α and IFN-γ in the blood detected by Elisa kit based on CT-26 model.

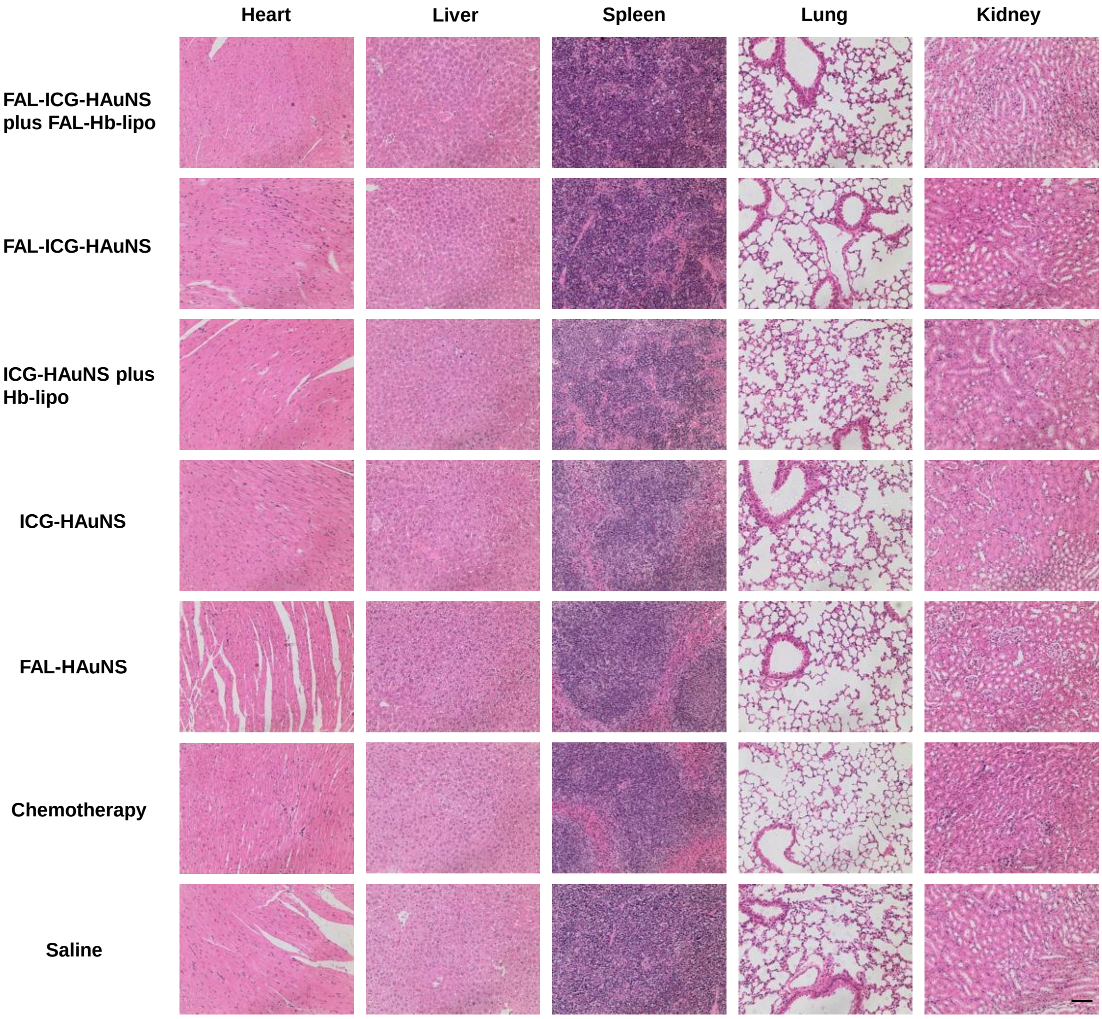

Supplementary Figure 13. Safety study of B16 tumor model. Representative H&E staining photographs of heart, liver, spleen, lung and kidney in each group. Scale bars, 100 μm.

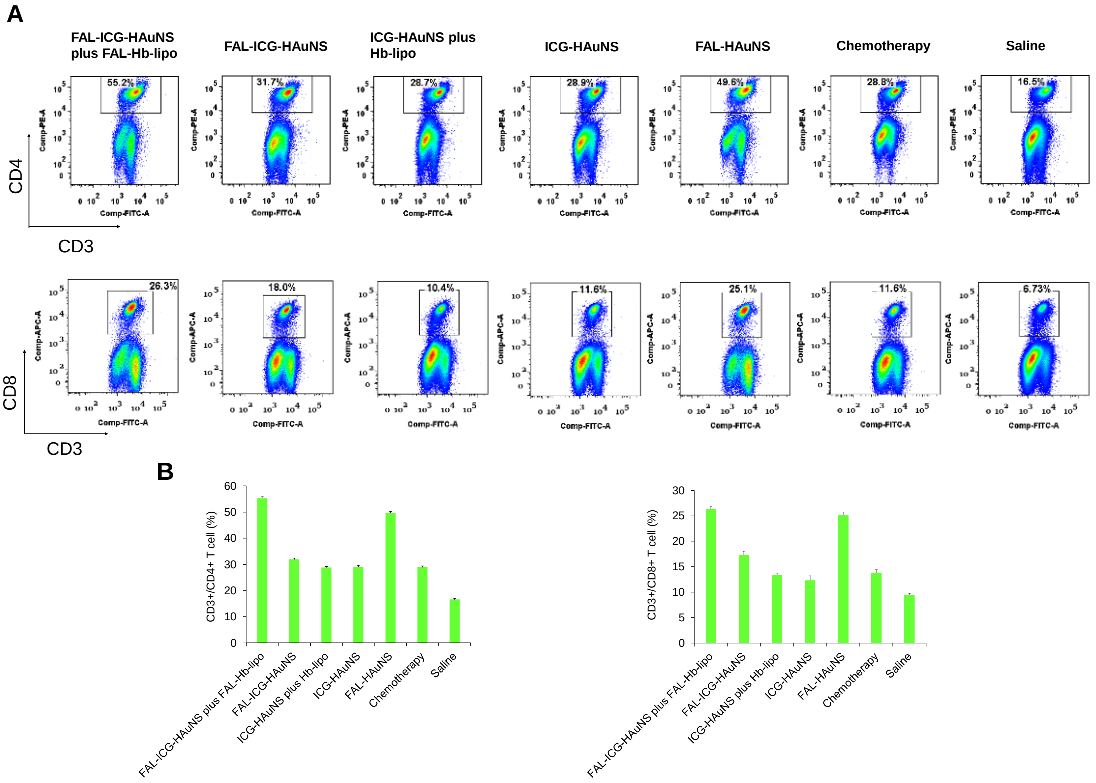

Supplementary Figure 14. Immune response of B16 tumor model. Representative flow cytometric plots of CD8^{+}, CD4^{+} T cells in splenic lymphocytes (A) and quantification (B) in different groups, n = 6. All error bars are expressed as ± SD.

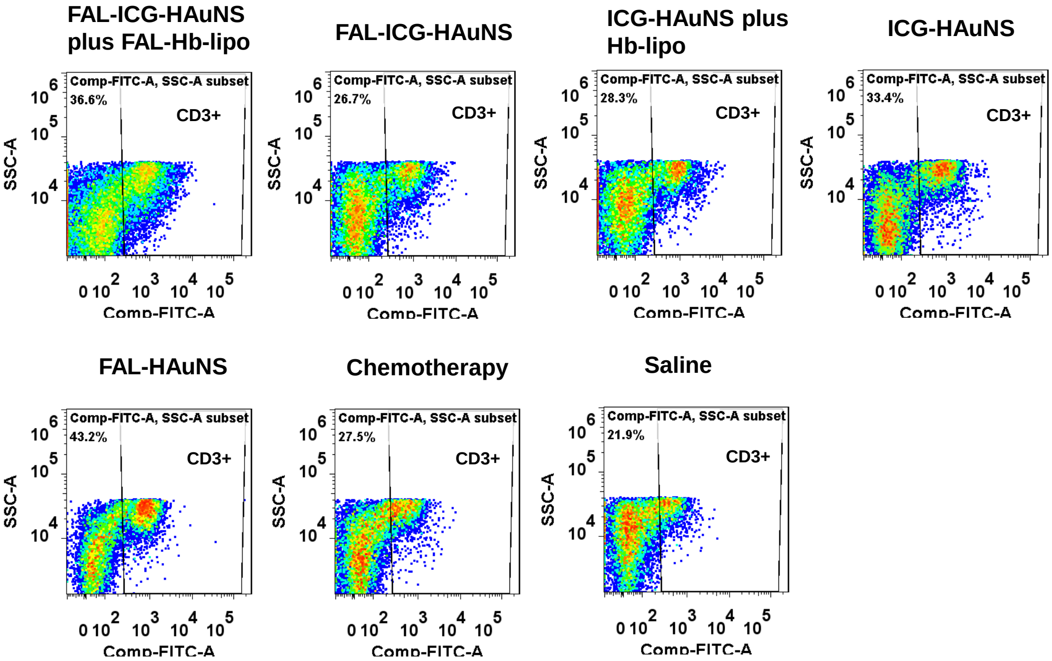

Supplementary Figure 15. Infiltration of CD3^{+} T cells. Infiltration of CD3^{+} T

cells in tumor tissue after anti-tumor treatment based on B16 model.

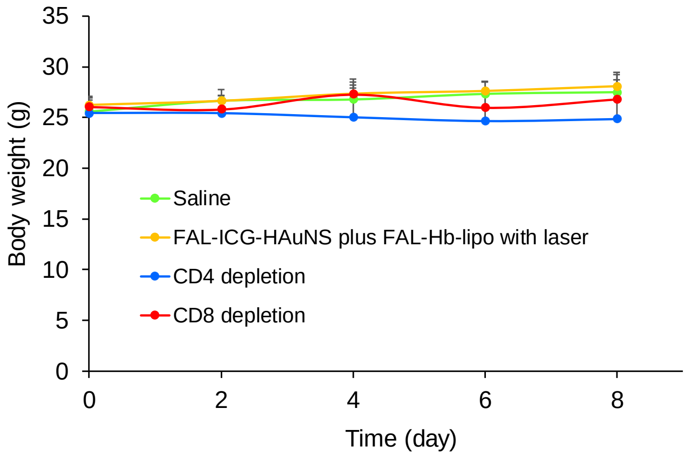

Supplementary Figure 16. Study of Body weight change. Body weight change curves of mice in the process of T cell depletion, n = 6. All error bars are expressed as ± SD.

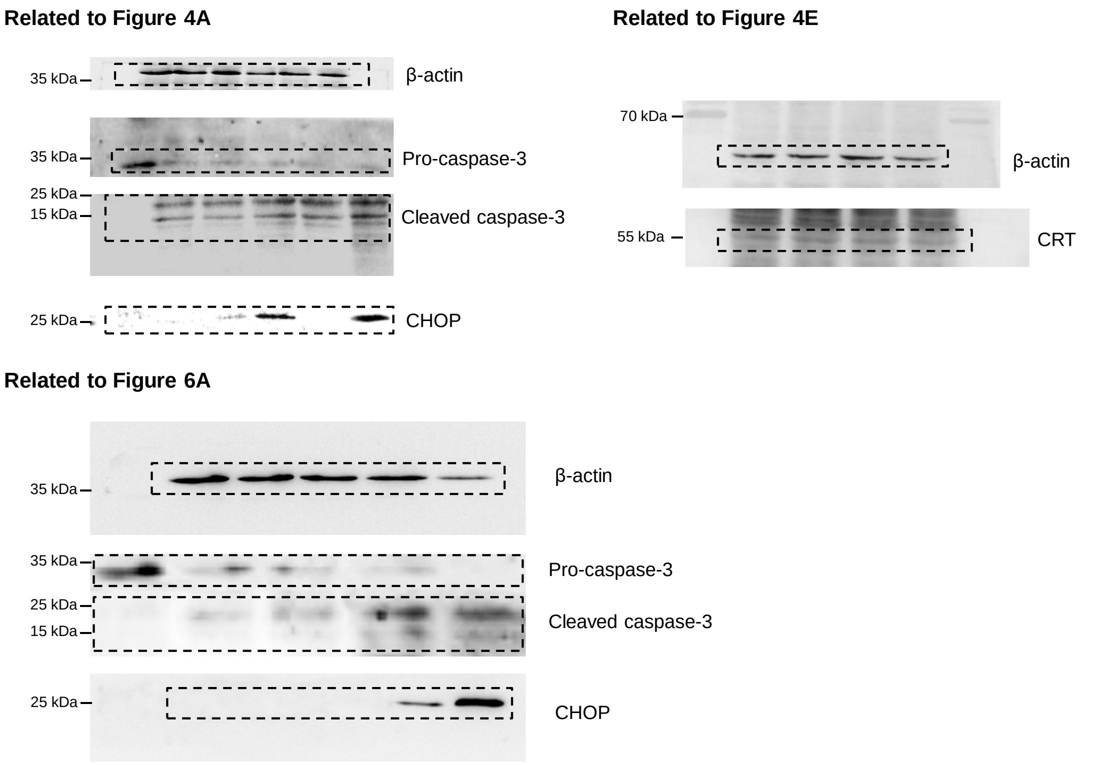

Supplementary Figure 17. Uncropped scants of western blots.
