# Supplementary material for: Targeting photodynamic and photothermal therapy to the endoplasmic reticulum enhances immunogenic cancer cell death
Source: Nat Commun. 2019 Jul 26;10:3349. doi: 10.1038/s41467-019-11269-8 (PMC6659660; doi:10.1038/s41467-019-11269-8)
Supplement: Supplementary file 2 — Reporting Summary [file 41467_2019_11269_MOESM2_ESM.pdf]

nature research

Corresponding author(s): Jian You

Reporting Summary

Nature Research wishes to improve the reproducibility of the work that we publish. This form provides structure for consistency and transparency in reporting. For further information on Nature Research policies, see Authors & Referees and the Editorial Policy Checklist.

Statistical parameters

When statistical analyses are reported, confirm that the following items are present in the relevant location (e.g. figure legend, table legend, main text, or Methods section).

n/a	Confirmed	
☐	☒	The exact sample size (n) for each experimental group/condition, given as a discrete number and unit of measurement
☐	☒	An indication of whether measurements were taken from distinct samples or whether the same sample was measured repeatedly
☐	☒	The statistical test(s) used AND whether they are one- or two-sided<br>*Only common tests should be described solely by name; describe more complex techniques in the Methods section.*
☐	☒	A description of all covariates tested
☐	☒	A description of any assumptions or corrections, such as tests of normality and adjustment for multiple comparisons
☐	☒	A full description of the statistics including central tendency (e.g. means) or other basic estimates (e.g. regression coefficient) AND variation (e.g. standard deviation) or associated estimates of uncertainty (e.g. confidence intervals)
☐	☒	For null hypothesis testing, the test statistic (e.g. F, t, r) with confidence intervals, effect sizes, degrees of freedom and P value noted<br>*Give P values as exact values whenever suitable.*
☐	☒	For Bayesian analysis, information on the choice of priors and Markov chain Monte Carlo settings
☐	☒	For hierarchical and complex designs, identification of the appropriate level for tests and full reporting of outcomes
☐	☒	Estimates of effect sizes (e.g. Cohen's d, Pearson's r), indicating how they were calculated
☐	☒	Clearly defined error bars<br>*State explicitly what error bars represent (e.g. SD, SE, CI)*

Our web collection on statistics for biologists may be useful.

Software and code

Policy information about availability of computer code

Data collection	No software was used.
Data analysis	Image J and FlowJo were used.

For manuscripts utilizing custom algorithms or software that are central to the research but not yet described in published literature, software must be made available to editors/reviewers upon request. We strongly encourage code deposition in a community repository (e.g. GitHub). See the Nature Research guidelines for submitting code & software for further information.

Data

Policy information about availability of data

All manuscripts must include a data availability statement. This statement should provide the following information, where applicable:

- Accession codes, unique identifiers, or web links for publicly available datasets
- A list of figures that have associated raw data
- A description of any restrictions on data availability

All data generated or analysed during this study are included in this published article (and its supplementary information files).

Field-specific reporting

Please select the best fit for your research. If you are not sure, read the appropriate sections before making your selection.

- [x] Life sciences
- [ ] Behavioural & social sciences
- [ ] Ecological, evolutionary & environmental sciences

For a reference copy of the document with all sections, see nature.com/authors/policies/ReportingSummary-flat.pdf

Life sciences study design

All studies must disclose on these points even when the disclosure is negative.

Sample size	According to relevant studies, the sample size of the cytotoxicity in vitro is 5 (n=5). The sample size of the ROS generation and western blotting experiment is 3 (n=3). The the sample size of animal experiment is 5 (n=5). The sample size of Flow cytometry assay is 3 (n = 3).
Data exclusions	No data were excluded from the analyses.
Replication	In the case of the same experimental materials, experimental conditions and experimental method, one experiment was repeated three times or more by the same individual operation. All the attempts at replication were successful.
Randomization	Experimental animals were randomly divided into several groups by random block method according to body weight, n = 6.
Blinding	The investigators were blinded to group allocation during data collection and analysis.

Reporting for specific materials, systems and methods

Materials & experimental systems

n/a	Involved in the study	
☐	☐	Unique biological materials
☐	☒	Antibodies
☐	☒	Eukaryotic cell lines
☐	☐	Palaeontology
☐	☒	Animals and other organisms
☐	☐	Human research participants

Methods

n/a	Involved in the study	
☐	☐	ChIP-seq
☐	☒	Flow cytometry
☐	☐	MRI-based neuroimaging

Unique biological materials

Policy information about availability of materials

Obtaining unique materials	*Describe any restrictions on the availability of unique materials OR confirm that all unique materials used are readily available from the authors or from standard commercial sources (and specify these sources).*

Antibodies

Antibodies used

CHOP (L63F7) Mouse mAb #2895 from Cell signaling technology, catalog # 2895, colon name L63F7, lot number 11
MHC Class I (H-2Kd) Monoclonal Antibody (SF1-1.1.1), PE, eBioscience™ from Thermo Fisher Scientific, catalog # 12-5957-82, RRID AB_2043875.
MHC Class II (I-A/I-E) Monoclonal Antibody (M5/114.15.2), FITC, eBioscience™ from Thermo Fisher Scientific, catalog # 11-5321-82, RRID AB_465232.
β-actin Monoclonal Antibody from Proteintech Group, catalog # 60008-1-Ig, colon name 7D2C10
CRT Rabbit Polyclonal Antibody form Proteintech Group, catalog # 10292-1-AP
Anti-active Caspase-3 antibody from Abcam, catalog # ab214430, colon name EPR21032, lot number GR3212358-3
FITC anti-mouse CD3 antibody from Biolegend (California, MA), catalog # 100203, clone name 17A2, lot number B261318
PE anti-mouse CD4 antibody from Biolegend (California, MA), catalog #100407, clone name GK1.5, lot number B233413
PE anti-mouse IFN-γ antibody from Biolegend (California, MA), catalog # 505807, clone name XMG1.2, lot number B265788
APC anti-mouse CD8α antibody from Biolegend (California, MA),catalog # 100712, clone name 53-6.7, lot number B244174
APC anti-mouse Foxp3 antibody from Lian Ke Biotechnology Co., Ltd. (Hangzhou, China), catalog # AM0F05-50, colon name 3G3, lot number A88970734

InVivomAb anti-mouse CD8α (clone 53-6.7) from BioXcell (West Lebanon, NH), catalog # BE0061
InVivomAb anti-mouse CD4 (cloneGK1.5) from BioXcell (West Lebanon, NH), catalog # BE0003-1

Validation

CHOP (L63F7) Mouse mAb #2895: https://www.cst-c.com.cn/products/primary-antibodies/chop-l63f7-mouse-mab/2895?site-search-type=Products&N=4294956287&Ntt=chop&fromPage=plp
Anti-active Caspase-3 antibody : https://www.abcam.com/active-caspase-3-antibody-epr21032-ab214430.html
MHC Class I (H-2Kd) Monoclonal Antibody (SF1-1.1.1), PE, eBioscience™ : https://www.thermofisher.com/antibody/product/MHC-Class-I-H-2Kd-Antibody-clone-SF1-1-1-1-Monoclonal/12-5957-82
MHC Class II (I-A/I-E) Monoclonal Antibody (M5/114.15.2), FITC, eBioscience™ : https://www.thermofisher.com/antibody/product/MHC-Class-II-I-A-I-E-Antibody-clone-M5-114-15-2-Monoclonal/11-5321-82
β-actin Monoclonal Antibody : http://www.ptgcn.com/products/ACTB-Antibody-60008-1-Ig.htm
CRT Rabbit Polyclonal Antibody : http://www.ptgcn.com/products/CALR-Antibody-10292-1-AP.htm
FITC anti-mouse CD3 antibody: https://www.biolegend.com/en-us/products/fitc-anti-mouse-cd3-antibody-45
PE anti-mouse CD4 antibody: https://www.biolegend.com/en-us/products/pe-anti-mouse-cd4-antibody-250
PE anti-mouse IFN-γ antibody: https://www.biolegend.com/en-us/products/pe-anti-mouse-ifn-gamma-antibody-997
APC anti-mouse CD8α antibody: https://www.biolegend.com/en-us/products/apc-anti-mouse-cd8a-antibody-150
APC anti-mouse Foxp3 antibody: http://www.liankebio.com/Web2004V2/datasheet/lianke/AM0F05.pdf
InVivomAb anti-mouse CD8α: https://bxcell.com/product/m-cd8a-2/
InVivomAb anti-mouse CD4: https://bxcell.com/product/m-cd4/

Eukaryotic cell lines

Policy information about cell lines

Cell line source(s)

CT-26 and B16 were purchased from Chinese Academy of Sciences Cell Bank, Shanghai.

Authentication

DNA was extracted from the CT-26 and B16 cell pellet using the Qiagen® QIAamp® Mini Kit, and the extracted DNA was amplified with SGMPlius®, then subjected to polyacrylamide gel electrophoresis using an ABI377 DNA sequencer. Then the results were analyzed by GeneScan® and Genotyper® software.

Mycoplasma contamination

All cell lines tested negative for mycoplasma contamination.

Commonly misidentified lines
(See ICLAC register)

This study did involve commonly misidentified lines.

Palaeontology

Specimen provenance

Provide provenance information for specimens and describe permits that were obtained for the work (including the name of the issuing authority, the date of issue, and any identifying information).

Specimen deposition

Indicate where the specimens have been deposited to permit free access by other researchers.

Dating methods

If new dates are provided, describe how they were obtained (e.g. collection, storage, sample pretreatment and measurement), where they were obtained (i.e. lab name), the calibration program and the protocol for quality assurance OR state that no new dates are provided.

☐ Tick this box to confirm that the raw and calibrated dates are available in the paper or in Supplementary Information.

Animals and other organisms

Policy information about studies involving animals; ARRIVE guidelines recommended for reporting animal research

Laboratory animals

All animals in this experiment were male Babl/c mice (six to eight weeks-old).

Wild animals

This study did not involve wild animals.

Field-collected samples

This study did not involve samples collected from the field.

Human research participants

Policy information about studies involving human research participants

Population characteristics

Describe the covariate-relevant population characteristics of the human research participants (e.g. age, gender, genotypic information, past and current diagnosis and treatment categories). If you filled out the behavioural & social sciences study design questions and have nothing to add here, write "See above."

Recruitment

Describe how participants were recruited. Outline any potential self-selection bias or other biases that may be present and how these are likely to impact results.

ChIP-seq

Data deposition

☐ Confirm that both raw and final processed data have been deposited in a public database such as GEO.

☐ Confirm that you have deposited or provided access to graph files (e.g. BED files) for the called peaks.

Data access links<br>*May remain private before publication.*	*For "Initial submission" or "Revised version" documents, provide reviewer access links. For your "Final submission" document, provide a link to the deposited data.*
Files in database submission	*Provide a list of all files available in the database submission.*
Genome browser session<br>(e.g. UCSC)	*Provide a link to an anonymized genome browser session for "Initial submission" and "Revised version" documents only, to enable peer review. Write "no longer applicable" for "Final submission" documents.*

Methodology

Replicates	*Describe the experimental replicates, specifying number, type and replicate agreement.*
Sequencing depth	*Describe the sequencing depth for each experiment, providing the total number of reads, uniquely mapped reads, length of reads and whether they were paired- or single-end.*
Antibodies	*Describe the antibodies used for the ChIP-seq experiments; as applicable, provide supplier name, catalog number, clone name, and lot number.*
Peak calling parameters	*Specify the command line program and parameters used for read mapping and peak calling, including the ChIP, control and index files used.*
Data quality	*Describe the methods used to ensure data quality in full detail, including how many peaks are at FDR 5% and above 5-fold enrichment.*
Software	*Describe the software used to collect and analyze the ChIP-seq data. For custom code that has been deposited into a community repository, provide accession details.*

Flow Cytometry

Plots

Confirm that:

☒ The axis labels state the marker and fluorochrome used (e.g. CD4-FITC).

☒ The axis scales are clearly visible. Include numbers along axes only for bottom left plot of group (a 'group' is an analysis of identical markers).

☒ All plots are contour plots with outliers or pseudocolor plots.

☒ A numerical value for number of cells or percentage (with statistics) is provided.

Methodology

Sample preparation	Mice were sacrificed with the whole tumors, spleen or lymph nodes isolated and minced using surgical scissors. Tissues were digested using Collagenase II and then strained through a 40 mm filter. After three rounds of PBS washes, single-cell suspensions were harvested and then subjected to fluorescein conjugated staining. For intracellular staining (such as IFN-γ and Foxp3), samples were incubated with penetration buffer BD Cytofix/Cytoperm kit according to manufacturer's protocol before adding antibodies. The preliminary FSC/SSC gates of the starting cell population was set according to the size of lymphocytes. All samples were subject to flow cytometry and analyzed using FlowJo software.
Instrument	BECKMAN COULTER, Cytomic FC 500MCL
Software	flowjo 7.6.1
Cell population abundance	The abundance of the relevant cell population within post-sort fractions is about 5000. The purity of the samples is above 90%. After sorting, samples are taken from the obtained samples, and then only analyzed by flow cytometry of the same settings parameters and same gate. And the positive ratio is the purity.
Gating strategy	The preliminary FSC/SSC gates of the starting cell population was set according to the size of lymphocytes. The staining cell populations were defined as positive when FL1 log is larger than 5.3 and FL2 log is larger than 4.4.

☐ Tick this box to confirm that a figure exemplifying the gating strategy is provided in the Supplementary Information.

Magnetic resonance imaging

Experimental design

Design type	*Indicate task or resting state; event-related or block design.*
Design specifications	*Specify the number of blocks, trials or experimental units per session and/or subject, and specify the length of each trial or block (if trials are blocked) and interval between trials.*
Behavioral performance measures	*State number and/or type of variables recorded (e.g. correct button press, response time) and what statistics were used to establish that the subjects were performing the task as expected (e.g. mean, range, and/or standard deviation across subjects).*

Acquisition

Imaging type(s)	*Specify: functional, structural, diffusion, perfusion.*
Field strength	*Specify in Tesla*
Sequence & imaging parameters	*Specify the pulse sequence type (gradient echo, spin echo, etc.), imaging type (EPI, spiral, etc.), field of view, matrix size, slice thickness, orientation and TE/TR/flip angle.*
Area of acquisition	*State whether a whole brain scan was used OR define the area of acquisition, describing how the region was determined.*

Diffusion MRI ☐ Used ☐ Not used

Preprocessing

Preprocessing software	*Provide detail on software version and revision number and on specific parameters (model/functions, brain extraction, segmentation, smoothing kernel size, etc.).*
Normalization	*If data were normalized/standardized, describe the approach(es): specify linear or non-linear and define image types used for transformation OR indicate that data were not normalized and explain rationale for lack of normalization.*
Normalization template	*Describe the template used for normalization/transformation, specifying subject space or group standardized space (e.g. original Talairach, MNI305, ICBM152) OR indicate that the data were not normalized.*
Noise and artifact removal	*Describe your procedure(s) for artifact and structured noise removal, specifying motion parameters, tissue signals and physiological signals (heart rate, respiration).*
Volume censoring	*Define your software and/or method and criteria for volume censoring, and state the extent of such censoring.*

Statistical modeling & inference

Model type and settings	*Specify type (mass univariate, multivariate, RSA, predictive, etc.) and describe essential details of the model at the first and second levels (e.g. fixed, random or mixed effects; drift or auto-correlation).*
Effect(s) tested	*Define precise effect in terms of the task or stimulus conditions instead of psychological concepts and indicate whether ANOVA or factorial designs were used.*

Specify type of analysis: ☐ Whole brain ☐ ROI-based ☐ Both

Statistic type for inference (See Eklund et al. 2016)	*Specify voxel-wise or cluster-wise and report all relevant parameters for cluster-wise methods.*
Correction	*Describe the type of correction and how it is obtained for multiple comparisons (e.g. FWE, FDR, permutation or Monte Carlo).*

Models & analysis

n/a	Involved in the study
☐	☐ Functional and/or effective connectivity
☐	☐ Graph analysis
☐	☐ Multivariate modeling or predictive analysis

Functional and/or effective connectivity	*Report the measures of dependence used and the model details (e.g. Pearson correlation, partial correlation, mutual information).*
Graph analysis	*Report the dependent variable and connectivity measure, specifying weighted graph or binarized graph, subject- or group-level, and the global and/or node summaries used (e.g. clustering coefficient, efficiency, etc.).*

Multivariate modeling and predictive analysis | *Specify independent variables, features extraction and dimension reduction, model, training and evaluation metrics.*
